# SARS‐CoV‐2 Spike Peptides Trigger Nociceptive Responses Through Spinal TLR4 Pathways

**DOI:** 10.1002/ejp.70343

**Published:** 2026-08-04

**Authors:** Bruno Eduardo Silva, Rayner Ribeiro Cardoso, Lívia Maria Ribeiro Rosário, João Paulo Prado, Rafaela Silva dos Santos, Flávio Protasio Veras, Mylena de Souza, Eduardo Maffud Cilli, Danilo Olivier, Marco Antonio de Andrade Belo, Ives Charlie‐Silva, Ester Siqueira Caixeta, Angel Roberto Barchuk, Albená Nunes‐Silva, Thiago Roberto Lima Romero, Giovane Galdino

**Affiliations:** ^1^ Laboratory of Neuroimmunobiology of Pain, Center for Experimental Biology Federal University of Alfenas Alfenas Minas Gerais Brazil; ^2^ Unesp – Institute of Chemistry – Araraquara Campus Sao Paulo Brazil; ^3^ Department of Physics and Interdisciplinary Technologies Federal University of Northern Tocantins Araguaína Brazil; ^4^ Brasil University Descalvado Sao Paulo Brazil; ^5^ Department of Cellular and Developmental Biology Federal University of Alfenas Alfenas Minas Gerais Brazil; ^6^ Insect Molecular Biology Laboratory Federal University of Alfenas Alfenas Minas Gerais Brazil; ^7^ Inflammation and Exercise Immunology Laboratory Federal University of Ouro Preto, Physical Education School Ouro Preto Minas Gerais Brazil; ^8^ Laboratory of Pain and Analgesia, Pharmacology Department Federal University of Minas Gerais Belo Horizonte Minas Gerais Brazil

**Keywords:** cytokines, microglia, pain, spike protein, toll‐like 4 receptors

## Abstract

**Background:**

Pain is a common neurological manifestation of COVID‐19, yet the mechanisms by which SARS‐CoV‐2 spike protein fragments contribute to nociceptive processing remain poorly understood. We investigated whether spike‐derived peptides directly activate spinal neuroimmune pathways involved in pain signalling.

**Methods:**

Male C57BL/6 mice received intrathecal administration of three synthetic SARS‐CoV‐2 spike‐derived peptides (PSPD2001, PSPD2002 or PSPD2003) or saline. Mechanical nociception was assessed using the von Frey test. The involvement of spinal Toll‐like receptor 4 (TLR4), microglia and p38 MAPK/NF‐κB signalling was investigated using pharmacological antagonists, TLR4 knockout mice, RT‐qPCR, ELISA, immunofluorescence, CX3CR1^GFP/+^ mice, human C20 microglial cells and molecular dynamics simulations.

**Results:**

All spike‐derived peptides induced mechanical nociception, with PSPD2003 producing the most pronounced response. PSPD2003 increased spinal TLR4 expression, elevated TNF‐α and IL‐6 levels and promoted activation of dorsal horn microglia, demonstrated by increased TMEM119‐ and CX3CR1‐positive cells. These nociceptive and neuroinflammatory effects were abolished by pharmacological inhibition of TLR4, microglia, p38 MAPK and NF‐κB signalling, as well as in TLR4^−/−^ mice, demonstrating that TLR4 signalling is essential for PSPD2003‐induced pain. PSPD2003 also induced a hypertrophic phenotype in human microglial cells, while molecular dynamics simulations supported a stable interaction with the TLR4/MD‐2 complex.

**Conclusions:**

These findings identify a previously unrecognized neuroimmune mechanism whereby a SARS‐CoV‐2 spike‐derived peptide triggers spinal nociception through TLR4‐dependent microglial activation and downstream p38 MAPK/NF‐κB signalling, highlighting the spinal TLR4–microglia axis as a potential therapeutic target for COVID‐19‐associated and post‐viral pain.

**Significance Statement:**

This study provides the first evidence that SARS‐CoV‐2 spike‐derived peptides directly activate a spinal TLR4‐dependent neuroimmune pathway to induce nociception. By integrating behavioural, pharmacological, genetic, cellular and computational approaches, it identifies microglial activation and p38 MAPK/NF‐κB signalling as key mechanisms linking viral peptides to pain, providing a mechanistic framework for COVID‐19‐ and post‐viral pain and supporting TLR4 as a potential therapeutic target.

## 1 Introduction

Since December 2019, millions of individuals worldwide have been affected by Coronavirus Disease 2019 (COVID‐19) (Liu et al. 2020). The International Committee on Taxonomy of Viruses designated the causative agent as Severe Acute Respiratory Syndrome Coronavirus 2 (SARS‐CoV‐2), which shares approximately 88% sequence identity with bat‐derived SARS‐related coronaviruses but exhibits lower homology with the original SARS‐CoV strain (ICTV 2020). Clinically, COVID‐19 commonly presents with fever, cough, myalgia and rhinorrhea and may progress to headache, dyspnoea, viral pneumonia and respiratory failure in severe cases (Li et al. 2020). Radiological findings frequently include alveolar infiltrates, fibrotic streaks, pleural effusion and hypoxemia (Zhou et al. 2020).

These clinical manifestations are closely linked to a dysregulated immune response known as the ‘cytokine storm’, characterized by excessive production of pro‐inflammatory cytokines following viral entry (Velavan and Meyer 2020). Elevated levels of interleukin‐6 (IL‐6), interleukin‐1β (IL‐1β) and tumour necrosis factor‐α (TNF‐α) promote immune cell infiltration, tissue injury and acute lung damage (Xie et al. 2020). Pain, particularly myalgia and arthralgia, is frequently reported in patients with COVID‐19 and represents a major contributor to early functional impairment (Lovell et al. 2020).

Pro‐inflammatory cytokines play a pivotal role in pain modulation at both peripheral and central levels. Peripherally, cytokines stimulate macrophages and monocytes to release prostaglandin E_2_, which sensitizes nociceptors and facilitates nociceptive transmission to the central nervous system (Jang et al. 2020). Centrally, cytokines activate spinal microglial cells, leading to the release of neuromodulators and inflammatory mediators that amplify nociceptive signalling (Song et al. 2024).

SARS‐CoV‐2 is an enveloped, single‐stranded RNA virus whose genome encodes structural proteins—spike (S), envelope (E), membrane (M) and nucleocapsid (N)—and nonstructural proteins essential for viral replication, including viral proteases and RNA‐dependent RNA polymerase (Lu et al. 2020; Chan et al. 2020; Chen et al. 2020). The spike protein is highly glycosylated and mediates viral attachment and entry through high‐affinity binding to the angiotensin‐converting enzyme 2 (ACE2) receptor, a process facilitated by the host transmembrane serine protease 2 (Letko et al. 2020; Fehr and Perlman 2015). Following entry, viral replication proceeds through the formation of the replicase–transcriptase complex, leading to virion assembly and release. Due to its central role in viral pathogenesis, the spike protein is a major target for antiviral strategies, including ACE2‐based peptides and protease inhibitors (Morse et al. 2020).

Toll‐like receptors, particularly Toll‐like receptor 4 (TLR4), have emerged as important mediators of SARS‐CoV‐2‐induced inflammation (Farooq et al. 2021; Farahani et al. 2022; Habeichi et al. 2024). TLR4 is a critical component of innate immunity, recognizing damage‐associated molecular patterns released during infection and tissue injury and amplifying inflammatory responses (Akira and Takeda 2004; Kawai and Akira 2007). Given its established role in sepsis and pneumonia, TLR4 may also contribute to COVID‐19‐associated pain. Based on this premise, the present study investigated the effects of three synthetic peptides (PSPD2001, PSPD2002 and PSPD2003), derived from the SARS‐CoV‐2 spike protein, on nociceptive threshold in mice, with a particular focus on the involvement of spinal TLR4.

## 2 Materials and Methods

### 2.1 Animals

The study adhered to the ARRIVE guidelines (Appendix S1). A total of 238 male C57BL/6 wild‐type, Toll‐like receptor 4 deficient (TLR4^−/−^) and CX3CR1^GFP/+^ mice (8 weeks old) were used. Animals were obtained from the local institutional vivarium.

TLR4^−/−^ mice, on a C57BL/6J background, are derived from well‐established strains originally developed and made available by The Jackson Laboratory (e.g., Stock No. 029015). CX3CR1^GFP/+^ mice correspond to the knock‐in strain B6.129P2(Cg)‐Cx3cr1^tm1Litt/J^, also available through The Jackson Laboratory (Stock No. 005582). In this strain, the CX3CR1 gene is partially replaced by a GFP reporter, resulting in disruption of receptor expression and enabling fluorescent identification of monocytes, microglia and dendritic cells. The genetic background and genotype of these strains were originally validated by The Jackson Laboratory.

Transgenic animals were genetically defined as follows: CX3CR1^GFP/+^ mice were maintained in the heterozygous state, whereas TLR4^−/−^ mice were homozygous knockout animals lacking functional TLR4 expression. These animals were kindly provided by Brazilian collaborators (University of São Paulo and Federal University of Minas Gerais) and subsequently maintained and bred in our institutional animal facility under specific pathogen‐free conditions.

Throughout the experiments, mice were housed in groups of up to six per cage with free access to food and water (ad libitum), under a 12 h light/dark cycle, at a controlled temperature of 22°C–24°C and relative humidity of 50% ± 5%. All experimental procedures were approved by the local Ethics Committee for the Use of Animals (protocol no. 0010/2021) and conducted in accordance with the guidelines of the International Association for the Study of Pain for the use of animals in research (Zimmermann 1983).

### 2.2 Synthesis, Purification and Characterization of Peptides

#### 2.2.1 Synthesis

To evaluate the nociceptive effects of peptides derived from the SARS‐CoV‐2 Spike protein, three peptides corresponding to distinct regions of the Spike S1 subunit were selected: PSPD2001 (RVYSSANNC; residues 158–166), PSPD2002 (VNLTTRT; residues 16–22) and PSPD2003 (NNATN; residues 121–125). The complete primary sequence of the SARS‐CoV‐2 Spike glycoprotein of the selected peptides is provided in Appendix S2. The compounds were synthesized using solid‐phase peptide synthesis based on the Fmoc (9‐fluorenylmethyloxycarbonyl) strategy. The chemical synthesis protocol consisted of iterative cycles of Fmoc deprotection and amino acid coupling, interspersed with multiple washes to remove excess reagents and by‐products.

Coupling reactions were carried out by activating the carboxyl groups of Fmoc‐protected amino acids in the presence of diisopropylcarbodiimide and hydroxybenzotriazole uronium for 2 h. A twofold molar excess of both Fmoc‐amino acids and coupling reagents relative to the number of reactive sites on the resin was used to maximize coupling efficiency. Following each coupling step, deprotection of the N‐terminal amino group was performed using a 20% solution of 4‐methylpiperidine in dimethylformamide. Completion of the deprotection reaction was monitored using a colorimetric ninhydrin test, which detects free amines based on a characteristic colour shift from yellow to violet‐blue after incubation at 110°C for 3 min (Behrendt et al. 2016). Between all reaction steps, thorough dimethylformamide washes were performed to ensure reaction purity and efficiency.

The resins employed in the synthesis included Fmoc‐Cys‐Wang, Fmoc‐Thr‐Wang and Fmoc‐Asn‐Wang, which enabled the preparation of peptides with a free C‐terminal carboxyl group. These resins were used to synthesize the following peptide sequences: RVYSSANNC‐COOH, VNLTTRT‐COOH and NNATN‐COOH.

#### 2.2.2 Cleavage

After completion of all coupling steps in the peptide sequences, the peptide chains were cleaved from the solid support by treatment with trifluoroacetic acid for 2 h. During cleavage, specific scavengers were added to protect amino acid side chains from acid‐mediated degradation and to prevent the reattachment of protecting groups, with the selection of scavengers tailored to each peptide sequence. Following cleavage, the peptides were precipitated by the addition of ice‐cold diethyl ether and subsequently extracted using a 0.045% trifluoroacetic acid solution in purified water, as previously described (Guy and Fields 1997). The peptide‐containing solutions were then lyophilized to obtain the crude peptide material in solid form.

#### 2.2.3 Purification

The crude peptides were purified by high‐performance liquid chromatography using a reversed‐phase column. Purification protocols were defined according to the retention times obtained during an analytical gradient run ranging from 5% to 95% acetonitrile over 30 min. Fractions corresponding to the purified peptides were collected, lyophilized and subsequently weighed to determine the synthesis yield, which was 13.0% for PSPD2001, 21.4% for PSPD2002 and 18.2% for PSPD2003 (Klaassen et al. 2019). The purified material was then subjected to chromatographic analysis to assess purity. Only samples with a purity of at least 95% were selected for biological assays, in accordance with quality standards established by the U.S. Food and Drug Administration (FDA). These regulatory guidelines stipulate that total contaminants must not exceed 5% of the sample, with no individual impurity surpassing 2.5%.

#### 2.2.4 Characterization

Purity analysis of the synthesized peptides was conducted using a Thermo LCQ Fleet mass spectrometer equipped with an electrospray ionization ion‐trap (ESI‐IT‐MS) system. Sample solutions were prepared at approximately 10 mg/L in a mixture of acetonitrile and water containing 0.1% (v/v) formic acid and were directly infused into the mass spectrometer. The infusion flow rate was maintained at 5.0 μL/min, and the electrospray source was operated in positive ion mode, with a voltage of 4.5 kV applied to the electrospray capillary. The resulting mass spectra provided molecular mass‐to‐charge (*m*/*z*) ratios corresponding to the analytes of interest, thereby confirming the successful synthesis and identity of the target peptides (Luna et al. 2016).

#### 2.2.5 Peptide Alignment

The similarities among the synthesized peptides (A) PSPD2001, (B) PSPD2002 and (C) PSPD2003 were evaluated using the CLUSTAL W programme version 1.83 (http://www.ebi.ac.uk/clustalw/). Sequence alignments were performed against entries deposited in the NCBI BLAST (Basic Local Alignment Search Tool) database, a suite of algorithms designed to identify local similarities between nucleotide or protein sequences. Searches were conducted in both nucleic acid and protein databases available at http://www.ncbi.nlm.nih.gov/blast. Specifically, protein BLAST (blastp) analyses were performed using the SwissProt protein sequence database, with taxonomy restricted to *Physalaemus cuvieri* (taxid: 218685). To obtain detailed annotations for proteins exhibiting sequence similarity with the peptides, the UniProtKB/SwissProt database (http://www.uniprot.org/) was also consulted.

### 2.3 Substances Used in the Study

In addition to peptide administration, several pharmacological agents were used to investigate the roles of TLR4, microglia, p38 MAP kinase and NF‐κB in the nociceptive process. The compounds included LPS‐RS (InvivoGen, USA), a TLR4 antagonist; minocycline (Sigma‐Aldrich, USA), a microglial inhibitor; SML0543 (Sigma‐Aldrich, USA), a p38 MAPK inhibitor; and PDTC (Sigma‐Aldrich, USA), an NF‐κB inhibitor. All compounds were diluted in sterile saline (0.9%), except PDTC, which was prepared in 2% dimethyl sulfoxide (DMSO). Doses were selected based on previously published studies (Wu et al. 2006; Chen et al. 2018; Elisei et al. 2020; Borghi et al. 2022).

### 2.4 Mechanical Nociceptive Threshold Measurement

To evaluate the nociceptive effects of the SARS‐CoV‐2 spike‐derived peptides (PSPD2001, PSPD2002 and PSPD2003), mechanical withdrawal thresholds were assessed using calibrated von Frey filaments (Aesthesio, USA; Stoelting, USA), following a standardized protocol routinely used in our laboratory. To evaluate nociceptive responses, animals were individually placed in darkened glass chambers positioned on an elevated metal mesh floor, which allowed access to the plantar surface of the hind paw. Animals were allowed to acclimate to the testing apparatus for 30–60 min on the day before testing and again on the experimental day before measurements were taken.

After acclimation, the filaments were applied perpendicularly to the plantar surface of the right hind paw in ascending order of force (0.02, 0.04, 0.07, 0.16, 0.4, 0.6, 1.0 and 1.4 g) until the filament bent slightly, indicating consistent force application. Each filament was applied for approximately 1–2 s. A positive nociceptive response was defined as a brisk paw withdrawal followed by licking or shaking.

Mechanical thresholds were determined using the ascending stimulus method and defined as the lowest filament force that evoked a positive response. To ensure response consistency, the filament force was considered valid only when it elicited withdrawal behaviour in consecutive applications. Each threshold determination consisted of a complete ascending series of filament applications and was performed independently three times per animal, with an interval of at least 3 min between determinations to avoid sensitization. The final mechanical withdrawal threshold for each animal was calculated as the mean of these three independent determinations (Elisei et al. 2020).

### 2.5 Induction of Nociception by SARS‐CoV‐2 Spike Protein‐Derived Peptides

To assess the spinal nociceptive effects of the peptides PSPD2001, PSPD2002 and PSPD2003, each peptide—or its vehicle (saline)—was administered intrathecally at two doses (25 and 50 ng). Baseline nociceptive thresholds were recorded for all animals prior to treatment. Following intrathecal injection, nociceptive thresholds were re‐evaluated at 1, 3, 5, 7 and 24 h.

### 2.6 Intrathecal Injection

As previously described, all peptides and pharmacological agents were administered via the intrathecal route. For this procedure, animals were anaesthetized with 2% isoflurane delivered in oxygen (2 L/min). The lumbar region was shaved and disinfected with 70% ethanol, and the animals were positioned in ventral decubitus to allow accurate identification of the L4–L5 intervertebral space. Intrathecal injections were performed at this site to access the subarachnoid space using a 13 × 0.3 mm needle, with a total injection volume of 5 μL. A characteristic tail flick or sudden tail movement was taken as an indicator of successful administration (Hylden and Wilcox 1980).

To standardize and validate the technique, a 2% lidocaine solution (Dentsply Pharmaceutical, USA) was intrathecally administered to a group of test animals, with the resulting transient hind limb paralysis confirming correct delivery into the intrathecal space. According to the experimental design, each pharmacological agent was administered 10 min prior to the corresponding peptide.

### 2.7 Experimental Protocol

Initially, to evaluate the nociceptive effects of the peptides PSPD2001, PSPD2002 and PSPD2003, mechanical nociceptive thresholds were assessed. Baseline measurements were obtained from each animal prior to the administration of each peptide or vehicle (0.9% sterile saline), and reassessed at 1, 3, 5, 7 and 24 h post‐administration. In experiments designed to investigate the spinal involvement of microglia, TLR4, p38 MAPK and NF‐κB, selective antagonists or inhibitors were administered intrathecally 10 min before each peptide injection (see Appendix S3 for details). Animals were randomly assigned in equal numbers to seven experimental groups (*n* = 6 per group) using a computer‐generated randomization sequence: PSPD2001 (25 μg), PSPD2001 (50 μg), PSPD2002 (25 μg), PSPD2002 (50 μg), PSPD2003 (25 μg), PSPD2003 (50 μg) and a vehicle group. All drug administrations and behavioural assessments were performed by investigators blinded to group allocation. In parallel, identical experimental groups were established using TLR4^−/−^ mice to determine the specific contribution of TLR4 to peptide‐induced nociception.

Additionally, to investigate the spinal involvement of TLR4, microglia, p38 MAPK and NF‐κB in peptide‐induced nociception, similar treatment groups were used in which animals were pretreated with the respective antagonists or inhibitors prior to peptide administration.

### 2.8 Quantitative Analysis of mRNA Expression Using RT‐PCR

Following euthanasia by isoflurane overdose at 3 h after intrathecal injection of each peptide at a 50 ng dose, spinal cord segments L4–L6 were promptly harvested, immersed in TRIzol reagent, and stored at −80°C until processing. Total RNA was extracted using the manufacturer's protocol, and RNA concentration and purity were determined with a NanoDrop 1000 spectrophotometer (Thermo Fisher Scientific, USA), with purity assessed by the A260/A280 ratio.

Complementary DNA (cDNA) was synthesized from total RNA using the High‐Capacity RNA‐to‐cDNA Reverse Transcription Kit (Thermo Fisher Scientific, USA). Quantitative real‐time PCR (qRT‐PCR) was performed to assess the expression of the mouse TLR4 gene, using SDHA as the endogenous control. Reactions were conducted in triplicate using Power SYBR Green Master Mix (Applied Biosystems, Thermo Fisher Scientific) on an ABI Prism 7500 Sequence Detection System (Applied Biosystems). Each 20 μL reaction contained 10 μL SYBR Green, 7 μL DNase/RNase‐free water, 1 μL of each primer (5 pmol) and 1 μL cDNA (25 ng). Amplification conditions consisted of 40 cycles of 10 s at 95°C followed by 1 min at 60°C.

Relative gene expression was quantified using the 2^ΔΔCt^ method (ΔΔCt = ΔCt_unknown − ΔCt_control) as described by Pfaffl (2001). Amplification efficiency for each gene was calculated using the LinRegPCR programme, following the recommendations of Ramakers et al. (2003). Primer sequences used for PCR amplification were: *Tlr4* forward 5′‐CCTGACACCAGGAAGCTTGAA‐3′, reverse 5′‐TCTGATCCATGCATTGGTAGGT‐3′; Sdha forward 5′‐GGAACACTCCAAAAACAGACCT‐3′, reverse 5′‐CCACCACTGGGTATTGAGTAGAA‐3′.

### 2.9 Quantification of Cytokine Levels

Considering that activation of the TLR4 signalling pathway promotes the production of pro‐inflammatory cytokines—key mediators involved in spinal nociceptive transmission—we quantified spinal TNF‐α and IL‐6 levels by enzyme‐linked immunosorbent assay (ELISA) 3 h after intrathecal administration of each peptide (50 ng) or vehicle. For this analysis, spinal cord segments (L4–L6) from each euthanized animal (*n* = 5 per group) were collected and transferred to microtubes containing 1 mL of phosphate‐buffered saline (PBS), followed by tissue homogenization at 3000 rpm for 10 min at 4°C. The resulting supernatants were transferred to 96‐well microplates, and cytokine concentrations were determined using a commercial ELISA kit (Prepotech, Cranbury, NJ, USA), according to the manufacturer's instructions. Absorbance readings were obtained using a microplate reader (ELX800, BioTek, USA), and data acquisition and analysis were performed with Gen5 software (BioTek, USA).

### 2.10 Immunofluorescence

Based on the hypothesis that SARS‐CoV‐2 S‐derived peptides activate TLR4—potentially expressed in spinal microglia and contributing to central sensitization—we investigated the effects of PSPD2003 in CX3CR1^GFP/+^, TLR4^−/−^, and wild‐type mice. Spinal cord slices from lumbar segments (L4–L6) were collected 3 h after intrathecal injection of PSPD2003 or vehicle. Prior to tissue collection, animals were anaesthetized intraperitoneally with 2.5% tribromoethanol (TBE, 0.1 mL/kg) and perfused transcardially with phosphate‐buffered saline (PBS, pH 7.4), followed by 4% paraformaldehyde (PFA) in 0.1 M PBS. Spinal cords were then carefully removed, post‐fixed in 4% PFA for 24 h, and transferred to a 30% sucrose solution in PBS for an additional 24 h for cryoprotection.

Coronal cryosections (20 μm) were obtained and washed in 0.1 M PBS. Sections were incubated for 2 h in a blocking solution containing 2% bovine serum albumin (BSA, Sigma‐Aldrich, USA) and 0.1% Triton X‐100 in 0.1 M PBS (pH 7.4). Following blocking, spinal cord sections from wild‐type mice were incubated overnight at 4°C with the primary antibody anti‐TMEM119 (1:100, Cell Signaling, USA), a well‐established microglial marker. The antibody was diluted in 0.1 M PBS with 0.1% Triton X‐100 and 2% BSA. After primary incubation, sections were washed three times in PBS and then incubated for 2 h at room temperature with an Alexa Fluor 488‐conjugated secondary antibody (1:200; Santa Cruz Biotechnology, USA), diluted in the same blocking buffer.

Following secondary antibody incubation, sections were washed in PBS and mounted on glass slides using Fluoromount‐G mounting medium containing DAPI (1:2000; SouthernBiotech, USA) for nuclear staining. Digital images were acquired using a K3 Nikon confocal fluorescence microscope (Japan) with a ×10 objective lens. Representative regions of the ipsilateral spinal cord dorsal horn (SCDH) were subsequently digitally enlarged to 60× magnification for detailed evaluation of immunofluorescence labeling and microglial morphology.

Immunofluorescence images were analysed using ImageJ software (National Institutes of Health, Bethesda, MD, USA). For quantitative analysis, a predefined region of interest (ROI) encompassing laminae II–IV of the ipsilateral spinal cord dorsal horn (SCDH) was delineated and applied uniformly to all images. Cell quantification was performed in one spinal cord section per animal (*n* = 3 animals per group), and the number of TMEM119‐positive or CX3CR1‐positive microglia within the ROI was manually counted by an investigator blinded to the experimental groups.

### 2.11 Molecular Dynamics Simulations Setup

To evaluate the temporal stability of the PSPD2003 peptide in complex with TLR4, a comprehensive molecular dynamics (MD) analysis was performed. All simulations were conducted using the AMBER24 software package (Case et al. 2024). The initial coordinates for the *Mus musculus* TLR4–MD‐2 complex were retrieved from the Protein Data Bank (PDB ID: 7MLM). The TLR4–MD‐2–PSPD2003 complex was constructed from docking‐derived poses, and the protonation states of titratable residues at pH 7.5 were assigned using the H++ web server (Gordon et al. 2005). The FF19SB all‐atom force field was applied to the protein, and the TIP3P water model was used to describe the solvent (Tian et al. 2020). The system was solvated in a truncated octahedral TIP3P water box extending 10 Å from any solute atom and neutralized with Na^+^ counterions.

Energy minimization consisted of two stages. First, a restrained minimization with positional restraints of 10.0 kcal/mol·Å^2^ on protein–peptide heavy atoms was performed using 5000 steps of steepest descent followed by 5000 steps of conjugate gradient minimization. This was followed by an unrestrained minimization of 10,000 steps. After minimization, the system was gradually heated from 10 to 310 K over 500 ps in the canonical (NVT) ensemble while maintaining restraints of 10 kcal/mol·Å^2^ on the protein. Subsequently, equilibration was performed for 5 ns in the isothermal–isobaric (NPT) ensemble with restraints progressively reduced from 10 kcal/mol·Å^2^ to zero.

Production MD simulations were performed for 100 ns per replica in the NVT ensemble without restraints. Temperature (310 K) and pressure (1 atm) were regulated using Langevin dynamics. SHAKE constraints were applied to all bonds involving hydrogen atoms, and hydrogen mass repartitioning (HMR) enabled the use of a 4‐fs integration time step. Long‐range electrostatic interactions were treated using the particle–mesh Ewald (PME) method with an 8‐Å cutoff (Darden et al. 1993). Two independent replicas with different initial velocity seeds were conducted for each system to ensure reproducibility.

#### 2.11.1 Molecular Dynamics Analysis

Trajectory processing and post‐simulation analyses were carried out using CPPTRAJ from the AmberTools25 suite (Case et al. 2005; Roe and Cheatham 2013). System equilibration and convergence were evaluated by monitoring the root‐mean‐square deviation (RMSD) and the radius of gyration (Rg) of Cα atoms. Protein structural flexibility was further assessed by calculating the root‐mean‐square fluctuation (RMSF) of Cα atoms on a residue‐by‐residue basis throughout the equilibrated segments of the trajectories.

Representative conformational states of the TLR4–PSPD2003 complex were identified using a k‐means clustering algorithm, employing cluster sizes ranging from 2 to 6. Clustering performance and robustness were benchmarked using both the Davies–Bouldin Index (DBI) and silhouette coefficients, ensuring reliable discrimination among conformational ensembles.

The interaction energy between PSPD2003 and TLR4 was estimated using the generalized Born GB‐Neck2 implicit solvent model (Nguyen et al. 2013). Binding free energies were computed using the molecular mechanics/generalized Born surface area (MM/GBSA) approach, applied to snapshots extracted from the stable portion of each trajectory, corresponding to the final 50 ns of the production MD simulations.

### 2.12 Microglial Cell Culture, Pharmacological Treatment, ELISA and Immunofluorescence Analyses

Human C20 microglial cells were seeded at a density of 2 × 10^5^ cells/mL in 24‐well plates containing glass coverslips (*n* = 3 independent experiments performed in triplicate) and maintained in Dulbecco's Modified Eagle Medium/Ham's F‐12 (DMEM/F12; Sigma‐Aldrich, USA) supplemented with 10% foetal bovine serum (FBS; Sigma‐Aldrich, USA) and 1% penicillin–streptomycin (Life Technologies, USA). Cells were cultured at 37°C in a humidified atmosphere containing 5% CO_2_.

After 24 h of stabilization, cells were divided into the following experimental groups: (i) Control group, treated with complete medium only; (ii) PSPD2003 group, stimulated with PSPD2003 peptide (1 ng/mL diluted in complete medium) for 6 h; and (iii) LPS‐RS + PSPD2003 group, pretreated with the TLR4 antagonist LPS‐RS for 2 h prior to PSPD2003 stimulation, which was maintained for an additional 6 h.

Following treatments, culture supernatants were collected for cytokine quantification, whereas cells adhered to coverslips were processed for immunofluorescence analyses. Tumour necrosis factor‐alpha (TNF‐α) levels in culture supernatants were quantified by ELISA according to the protocol previously described in Section 2.9.

For immunofluorescence analyses, cells were fixed with 4% paraformaldehyde (PFA) for 10 min immediately after supernatant collection. Subsequently, cells were washed with phosphate‐buffered saline (PBS), permeabilized with PBS containing 0.2% Tween‐20 (PBS‐T), and blocked with PBS supplemented with 1% bovine serum albumin (BSA). Cells were then incubated overnight at 4°C with rabbit anti‐Iba1 primary antibody (1:200; Wako, Japan). After washing, samples were incubated with Alexa Fluor 594‐conjugated secondary antibody and subsequently stained with DAPI (1:3000; Sigma‐Aldrich, USA) for 10 min to visualize cell nuclei.

Coverslips were mounted on glass slides, and fluorescent images were acquired using a K3 confocal fluorescence microscope (Nikon, Japan). Images were subsequently analysed using ImageJ software (NIH, USA).

### 2.13 Statistical Analysis

Data were expressed as the mean ± standard deviation (SD). An a priori sample size calculation was performed using G*Power software, based on effect size estimates derived from the study by Elisei et al. (2020), assuming a significance level (*α*) of 0.05 and a statistical power of 80% (1 − *β*). Behavioural nociceptive thresholds obtained using the von Frey test were analysed by two‐way repeated‐measures ANOVA, with treatment and time as factors, followed by Bonferroni's post hoc test when appropriate. Real‐time PCR data were analysed by one‐way ANOVA followed by Bonferroni's multiple‐comparison test. In vivo ELISA and immunofluorescence data were analysed using an unpaired Student's *t*‐test. In all cases, *p* values < 0.05 were considered statistically significant. Statistical analyses and graph generation were performed using GraphPad Prism software, version 8.0 (GraphPad Software Inc., San Diego, CA, USA).

## 3 Results

### 3.1 Nociception Induced by SARS‐CoV‐2 Spike‐Derived Peptides and the Spinal Involvement of TLR4 in This Process

**FIGURE 1:**
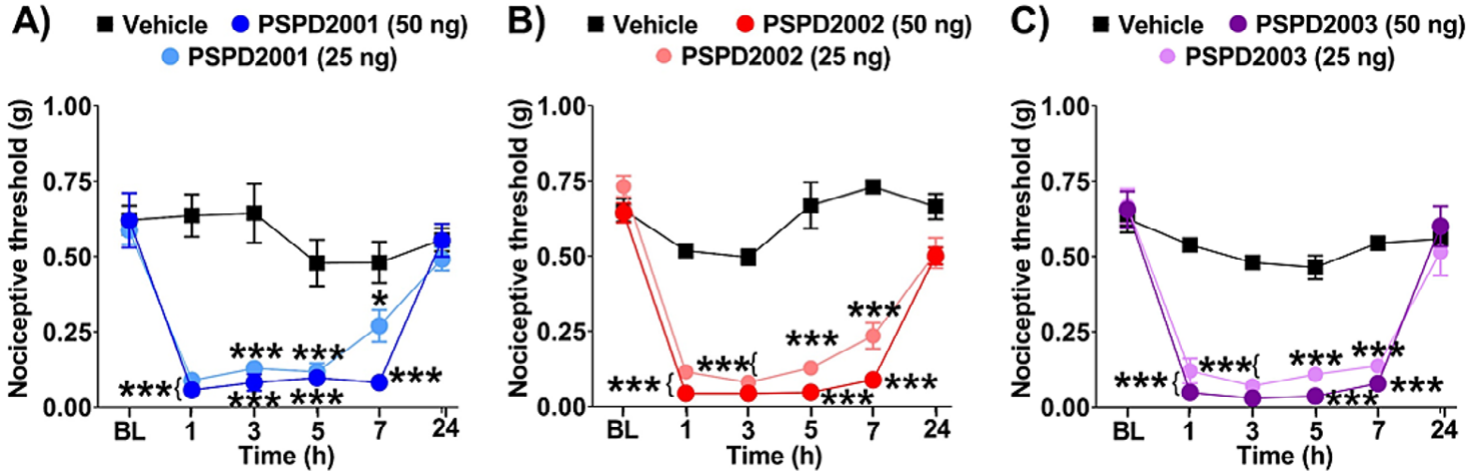
Assessment of the effects of intrathecal administration of SARS‐CoV‐2 S‐derived peptides PSPD2001 (25 and 50 μg; A), PSPD2002 (25 and 50 μg; B), and PSPD2003 (25 and 50 μg; C) on mechanical nociceptive thresholds in mice. Peptides were administered intrathecally, and nociceptive thresholds were evaluated at baseline (BL) and at 1, 3, 5, 7 and 24 h post‐injection. The vehicle group received 0.9% sterile saline. Data are presented as mean ± SD for 5–6 animals per group. *p < 0.05 and ***p < 0.001 indicate statistical significance compared with the vehicle‐treated group. Statistical analysis was performed using two‐way ANOVA followed by Bonferroni's multiple comparisons test.

Initially, the nociceptive effects of intrathecal administration of the peptides PSPD2001, PSPD2002 and PSPD2003 were evaluated at two different doses (25 ng and 50 ng). Two‐way ANOVA revealed a significant effect of treatment over time (*F*_5,12_ = 8.447, *p* < 0.001). As shown in Figure 1A, PSPD2001 significantly reduced the mechanical nociceptive threshold at both doses. At 25 ng, significant decreases were observed at 1 h (*p* < 0.001), 3 h (*p* < 0.001), 5 h (*p* < 0.001) and at 7 h (*p* < 0.05) compared with vehicle‐treated animals, whereas the 50 ng dose produced a more pronounced reduction in the mechanical nociceptive threshold from 1 to 7 h (*p* < 0.001). Similarly, PSPD2002 and PSPD2003 significantly induced mechanical hypersensitivity at both doses between 1 and 7 h after administration (Figure 1B,C; *p* < 0.001). No significant differences were detected at 24 h for any of the peptides. These findings demonstrate that intrathecal administration of SARS‐CoV‐2 S‐derived peptides induces transient mechanical hypersensitivity, supporting a nociceptive action at the spinal level.

**FIGURE 2:**
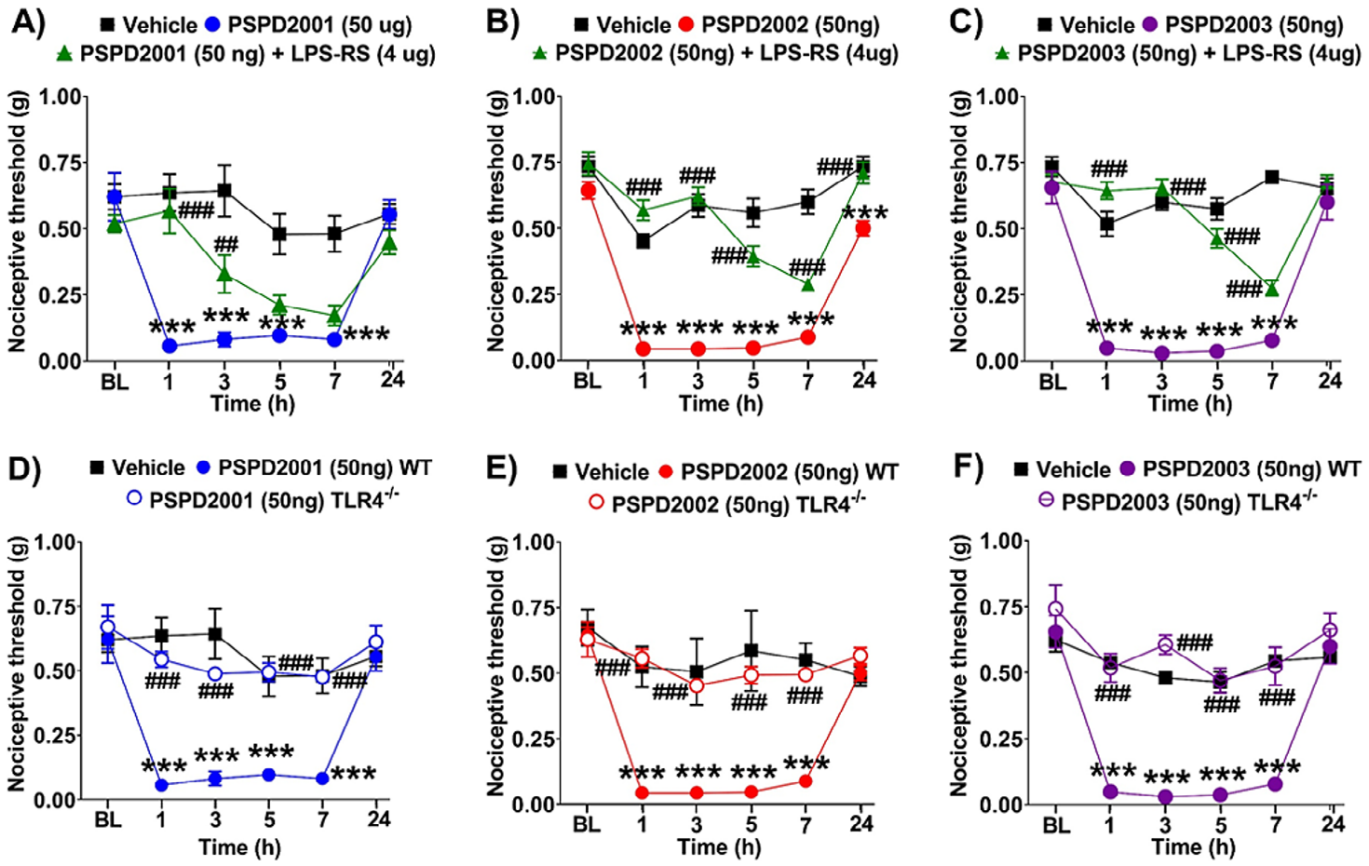
Investigation of the involvement of TLR4 in peptide‐induced nociception. Panels A–C show the effects of intrathecal pretreatment with the TLR4 antagonist LPS‐RS (4 μg), administered 10 min before intrathecal injection of SARS‐CoV‐2 S‐derived peptides PSPD2001 (A), PSPD2002 (B) or PSPD2003 (C), on mechanical nociceptive thresholds. Panels D–F show the corresponding effects of intrathecal administration of PSPD2001 (D), PSPD2002 (E) or PSPD2003 (F) in TLR4^−^/^−^ mice. Mechanical nociceptive thresholds were assessed at baseline (BL) and at 1, 3, 5, 7 and 24 h after peptide administration. Vehicle‐treated animals received 0.9% sterile saline. Data are presented as mean ± SD for 5–6 animals per group. ***p < 0.001 indicates statistical significance compared with the vehicle group; ##*p* < 0.01 and ###*p* < 0.001 indicate statistical significance compared with peptide‐treated groups. Statistical analysis was performed using two‐way ANOVA followed by Bonferroni's multiple comparisons test.

Moreover, the nociceptive responses elicited by the peptides were effectively reversed by prior intrathecal administration of the TLR4 antagonist LPS‐RS. Specifically, LPS‐RS significantly inhibited nociception induced by PSPD2001 at 1 h (*p* < 0.001; *F*_2,11_ = 7.666) and 3 h (*p* < 0.05; *F*_2,11_ = 7.666), by PSPD2002 from 1 h (*p* < 0.01; *F*_2,11_ = 13.10) through 5 h (*p* < 0.05; *F*_2,11_ = 13.10), and by PSPD2003 between 1 and 7 h post‐administration (*p* < 0.001; *F*_2,11_ = 9.37) (Figure 2A–C). Furthermore, peptide‐induced nociception was abolished in TLR4^−/−^ mice (Figure 2D–F), with none of the three peptides (PSPD2001, PSPD2002 and PSPD2003) eliciting any significant change in nociceptive threshold (*p* > 0.05). Collectively, these findings provide strong evidence supporting the role of TLR4 in mediating nociception induced by SARS‐CoV‐2 spike protein‐derived peptides evaluated in this study.

### 3.2 Spinal Microglia Contribute to Nociception Induced by SARS‐CoV‐2 Spike‐Derived Peptides

TLR4 is a key mediator in nociceptive responses, primarily by driving microglial activation, which contributes to central pain sensitization (Lacagnina et al. 2018). Therefore, after confirming the involvement of TLR4 in peptide‐induced nociception, the next step of the study was to evaluate the role of microglia in this process.

**FIGURE 3:**
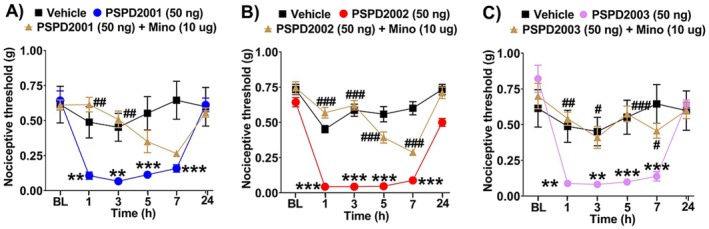
Evaluation of microglial involvement in peptide‐induced nociception. Panels A–C show the effects of intrathecal pretreatment with the microglial inhibitor minocycline (10 μg), administered 10 min before intrathecal injection of SARS‐CoV‐2 S‐derived peptides PSPD2001 (A), PSPD2002 (B) or PSPD2003 (C), on mechanical nociceptive thresholds. Mechanical thresholds were assessed at baseline (BL) and at 1, 3, 5, 7 and 24 h after peptide administration. The vehicle group received 0.9% sterile saline. Data are presented as mean ± SD for 5–6 animals per group. **p < 0.01 and ***p < 0.001 indicate statistical significance compared with the vehicle group; #*p* < 0.05, ##*p* < 0.01 and ###*p* < 0.001 indicate statistical significance compared with peptide‐treated groups. Statistical analysis was performed using two‐way ANOVA followed by Bonferroni's multiple comparisons test.

Figure 3A–C shows that pretreatment with the microglial activation inhibitor minocycline significantly reduced peptide‐induced nociception. This effect was observed from the first to the third hour in the PSPD2001 (50 ng) group (*p* < 0.001; *F*_2,11_ = 11.1), and from the first to the seventh hour in both the PSPD2002 (50 ng) (*p* < 0.001; *F*_2,11_ = 14.70) and PSPD2003 (50 ng) groups (*p* < 0.001; *F*_2,11_ = 10.34).

### 3.3 SARS‐CoV‐2 Peptides Modulate Spinal Pro‐Inflammatory Cytokine Levels

Given the evidence that microglial activation may contribute to SARS‐CoV‐2 peptide‐induced nociception, and considering that the release of pro‐inflammatory cytokines is a key mechanism underlying this response (Song et al. 2024), we next investigated the effects of each peptide on spinal TNF‐α and IL‐6 levels.

**FIGURE 4:**
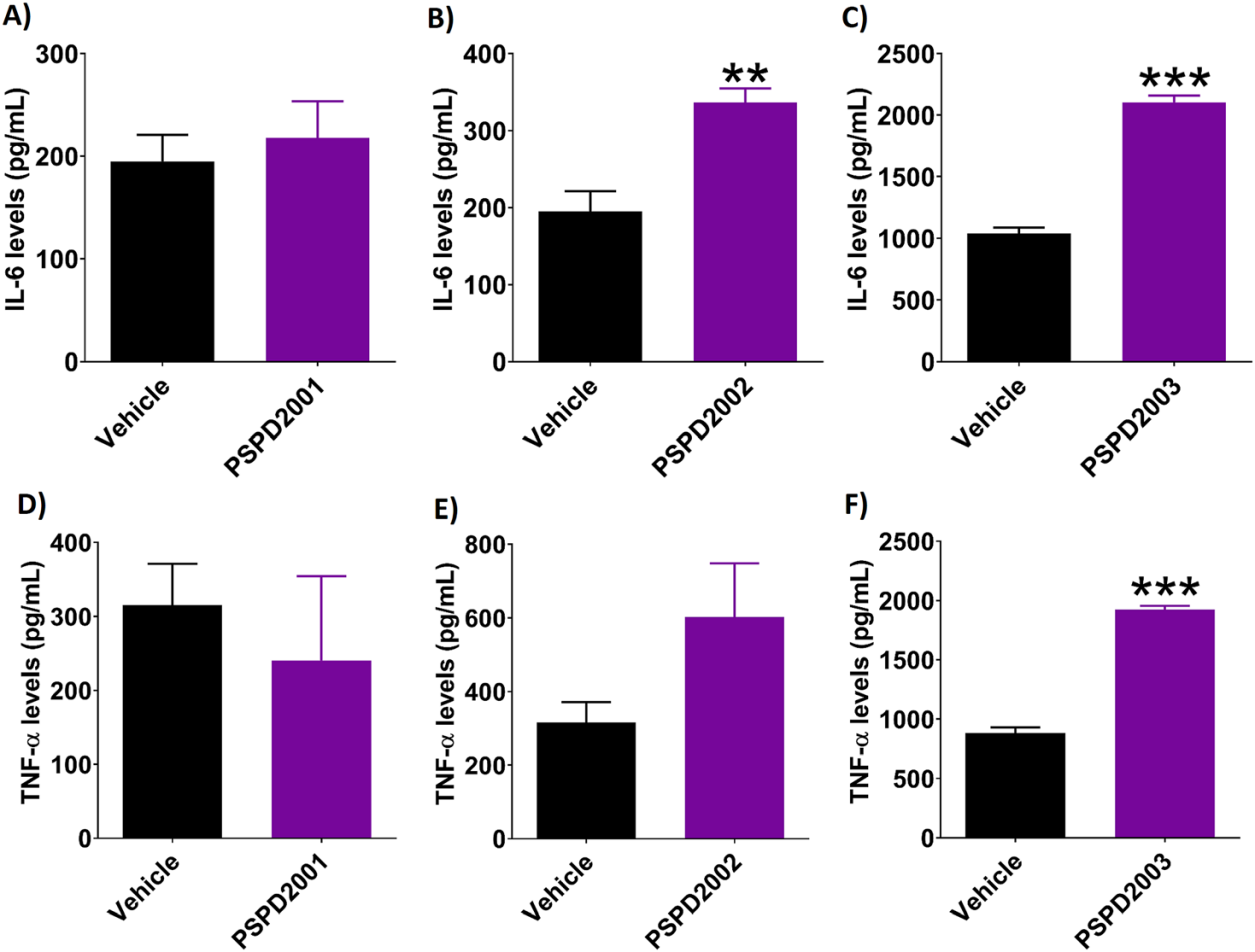
Effects of SARS‐CoV‐2‐derived peptides on spinal pro‐inflammatory cytokine levels. Spinal IL‐6 and TNF‐α levels were evaluated 3 h after intrathecal administration of PSPD2001, PSPD2002 or PSPD2003. Animals in the vehicle group received sterile saline solution (0.9%). Data are expressed as mean ± SD from 4–6 animals per group. **p < 0.01 and ***p < 0.001 indicate significant differences compared with the vehicle‐treated group. Statistical analyses were performed using one‐way ANOVA followed by Bonferroni's multiple comparisons test.

Three hours after intrathecal administration, PSPD2002 and PSPD2003 significantly increased spinal IL‐6 levels compared with the vehicle‐treated group (*p* < 0.01 and *p* < 0.001, respectively; *F*_3,10_ = 10.26; Figure 4B,C). Furthermore, PSPD2003 increased spinal TNF‐α levels compared with vehicle‐treated animals (*p* < 0.001; *F*_3,10_ = 10.26; Figure 4E).

### 3.4 PSPD2003 Increases Spinal TLR4 mRNA Expression

**FIGURE 5:**
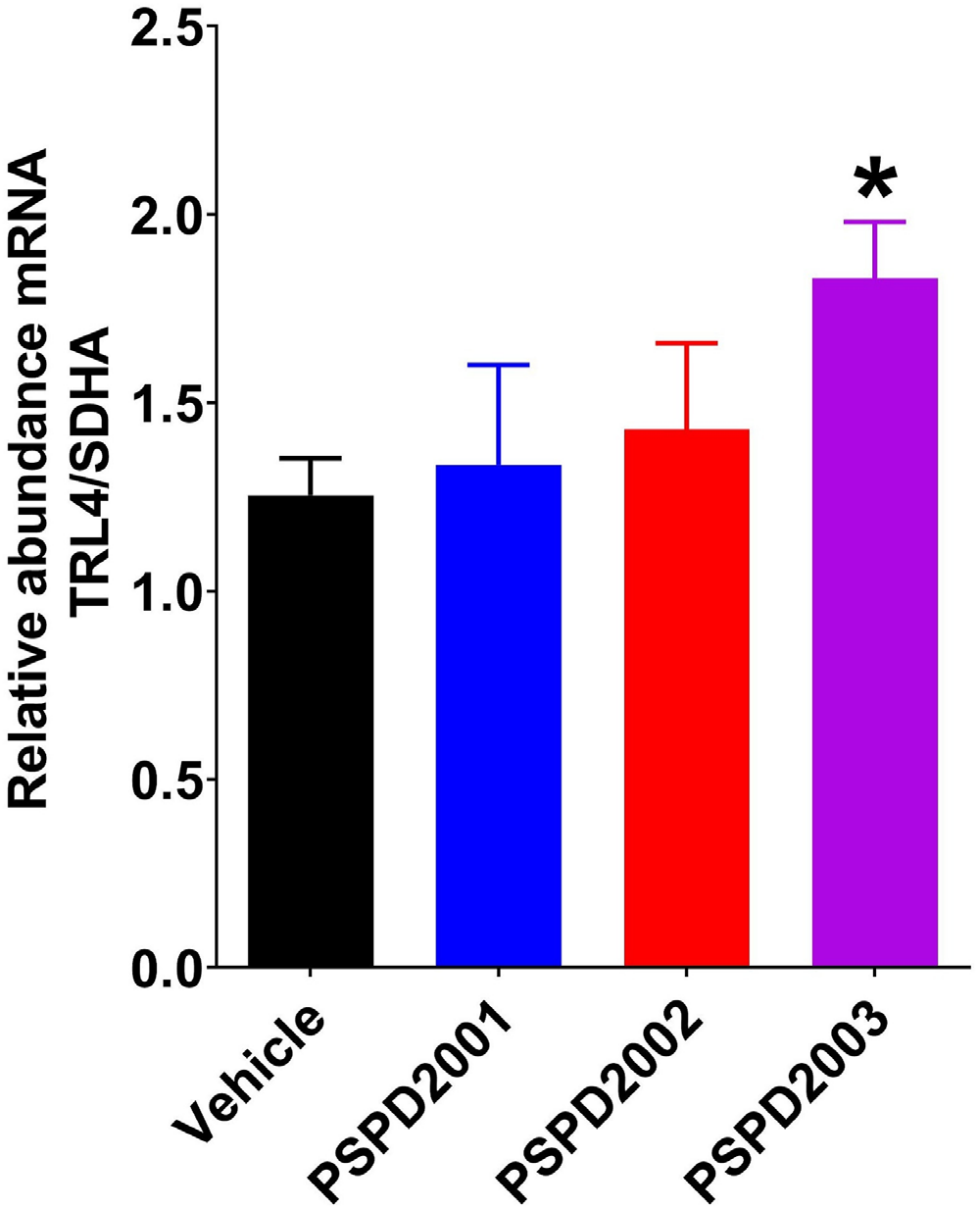
TLR4 mRNA expression in the spinal cord following intrathecal administration of SARS‐CoV‐2 S‐derived peptides. Mice received intrathecal injections of PSPD2001, PSPD2002 or PSPD2003 and TLR4 mRNA levels were quantified in spinal cord samples (L4–L6 segments) collected 3 h after peptide administration using RT‐qPCR and normalized to SDHA expression. The vehicle group received 0.9% sterile saline. Data are presented as mean ± SD for 4–5 animals per group. *p < 0.05 indicates statistical significance compared with the vehicle group. Statistical analysis was performed using one‐way ANOVA followed by Bonferroni's multiple comparisons test.

Given the behavioural findings indicating TLR4 involvement in peptide‐induced nociception, we next investigated the spinal mRNA expression of this receptor. Three hours after the intrathecal administration of PSPD2001, PSPD2002 or PSPD2003 (50 ng), a significant increase (*p* < 0.05; *F*_3,10_ = 2.268) in TLR4 mRNA expression was observed exclusively in the PSPD2003‐treated group (Figure 5).

In light of these findings, the subsequent experiments in this study were specifically designed to investigate the mechanisms underlying PSPD2003‐induced nociception.

### 3.5 The Intracellular p38 MAPK/NF‐κB Signalling Pathway Mediates PSPD2003‐Induced Nociception

Once activated by TLR4, spinal microglia initiate intracellular signalling cascades primarily through p38 MAPK, which subsequently activates transcription factors such as NF‐κB. This activation promotes the production and release of pro‐inflammatory cytokines, contributing to the sensitization of second‐order neurons and thereby facilitating the transmission of nociceptive signals (Zhang et al. 2023).

**FIGURE 6:**
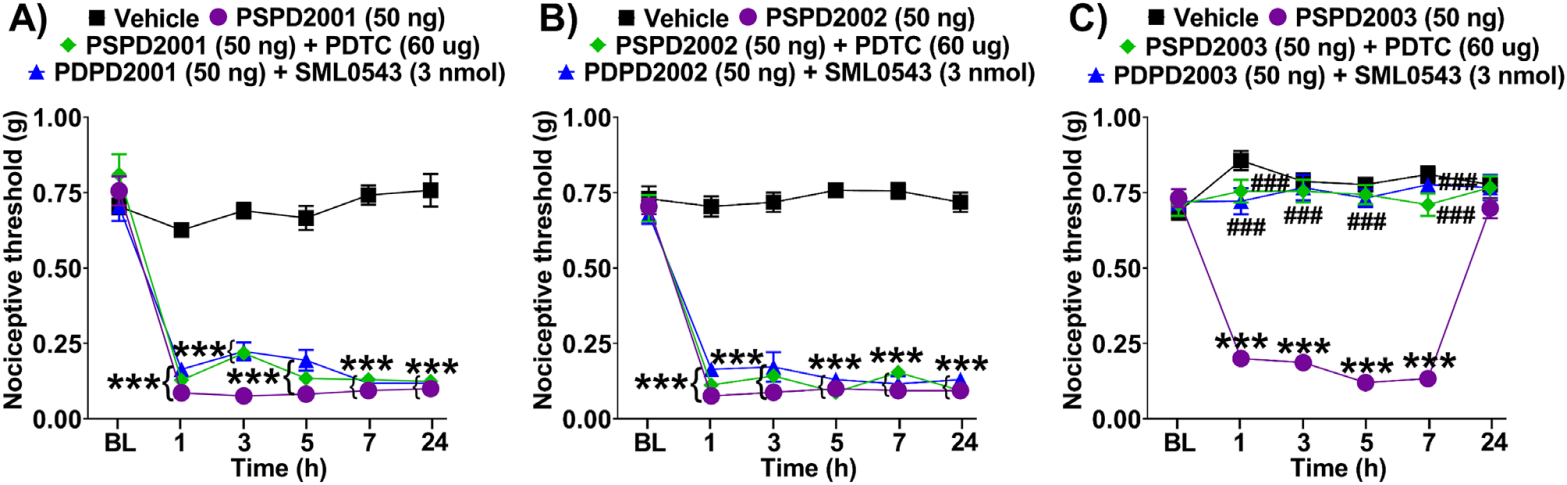
Evaluation of the involvement of p38 MAPK and NF‐κB signalling following administration of SARS‐CoV‐2 spike peptides. Mice received intrathecal pretreatment with the p38 MAPK inhibitor SML0543 or the NF‐κB inhibitor PDTC, administered 10 min before intrathecal injection of PSPD2001 (A), PSPD2002 (B) and PSPD2003 (C). Mechanical nociceptive thresholds were assessed at baseline (BL) and at 1, 3, 5, 7 and 24 h after peptide administration. Vehicle‐treated animals received 0.9% sterile saline (for SML0543) or 2% dimethyl sulfoxide (DMSO; for PDTC). Data are presented as mean ± SD for 5–6 animals per group. ***p < 0.001 indicates statistical significance compared with the respective vehicle‐treated groups; ###*p* < 0.001 indicates statistical significance compared with PSPD2003‐treated groups. Statistical analysis was performed using two‐way ANOVA followed by Bonferroni's multiple comparisons test.

Thus, to investigate the involvement of p38 MAPK and NF‐κB signalling in SARS‐CoV‐2 peptide‐induced nociception, animals were pretreated with the selective inhibitors SML0543 and PDTC, respectively. The results demonstrated that both SML0543 (3 nmol) and PDTC (60 μg) significantly reversed the nociceptive effects induced exclusively by PSPD2003 from 1 to 7 h after administration (*p* < 0.001; *F*_2,11_ = 17.01) (Figure 6C).

### 3.6 Immunofluorescent Identification of Microglia in the Dorsal Horn of the Spinal Cord During Nociception

To evaluate microglial expression in the SCDH following intrathecal (i.t.) administration of PSPD2003, and to investigate the involvement of TLR4 in this process, an immunofluorescence assay was performed using TLR4^−/−^ and CX3CR1^GFP/+^ mice.

**FIGURE 7:**
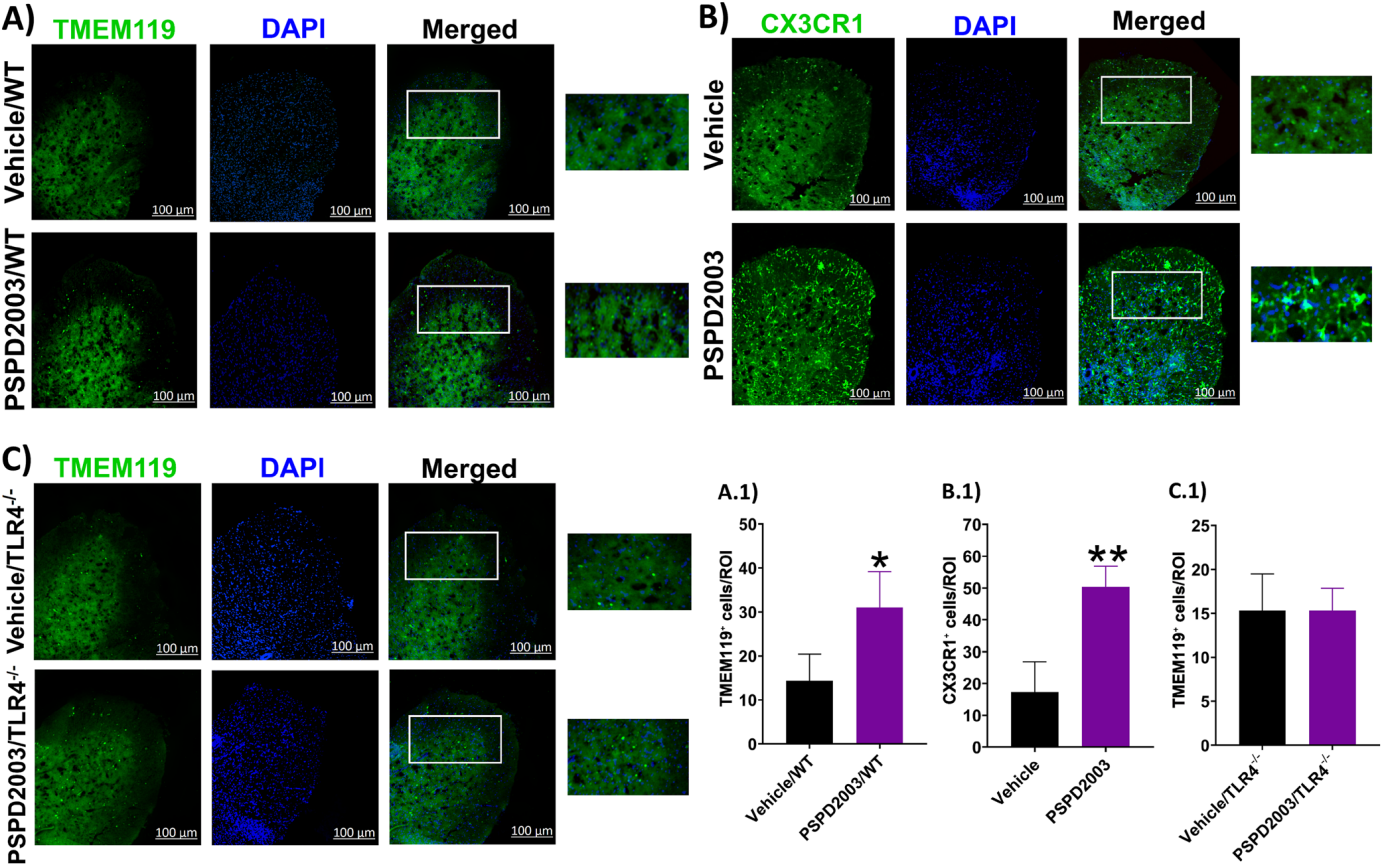
PSPD2003 induces TLR4‐dependent spinal microglial activation. Representative immunofluorescence and confocal images of the spinal cord dorsal horn (SCDH) obtained 3 h after intrathecal administration of PSPD2003 or vehicle. Fifty‐four sections from a total of 162 were randomly selected for image acquisition. Panels A and C show SCDH sections immunolabelled for TMEM119 (green), a marker of resident microglia, and counterstained with DAPI (blue). Panel B shows spinal cord sections from CX3CR1^GFP/+^ mice. Images were acquired using a 10× objective. Scale bar = 100 μm. Quantitative analyses (A.1–C.1) are presented as the mean ± SD of the number of TMEM119‐positive or CX3CR1‐positive microglia counted within a predefined region of interest (ROI) encompassing laminae II–IV of the ipsilateral SCDH. Cell quantification was performed in one spinal cord section per animal using ImageJ software (*n* = 3 animals per group). Statistical significance was determined using an unpaired Student's *t*‐test. *p* < 0.05 and **p < 0.001 versus the vehicle group.

As shown in Figure 7A,A.1, PSPD2003 administration significantly increased the number of TMEM119‐positive microglia in the SCDH 3 h after treatment compared with saline‐treated (vehicle) controls (*p* < 0.05). Similarly, the number of CX3CR1‐positive microglia was significantly increased in CX3CR1^GFP/+^ mice following PSPD2003 administration (Figure 7B,B.1; *p* < 0.01). Because TMEM119 is a specific marker of resident microglia and CX3CR1 is widely recognized as a marker associated with microglial activation, the concurrent increase in TMEM119‐ and CX3CR1‐positive cells provides complementary evidence that PSPD2003 induces spinal microglial activation.

Notably, the absence of an increase in the number of TMEM119‐positive microglia in the SCDH of TLR4^−^/^−^ mice (Figure 7C,C.1) suggests that PSPD2003‐induced microglial activation is mediated through TLR4‐dependent signalling pathways.

### 3.7 PSPD2003 Exhibits Stable and Specific Binding to TLR4

Following the behavioural and biomolecular evidence demonstrating the involvement of TLR4 in PSPD2003‐induced nociception, the present study employed a multifaceted approach, combining in silico MD simulations with functional in vivo assays to characterize the interaction between the SARS‐CoV‐2 Spike‐derived peptide PSPD2003 and TLR4. Overall, the findings demonstrate that PSPD2003 forms a stable and specific complex with TLR4 and modulates its activity.

**FIGURE 8:**
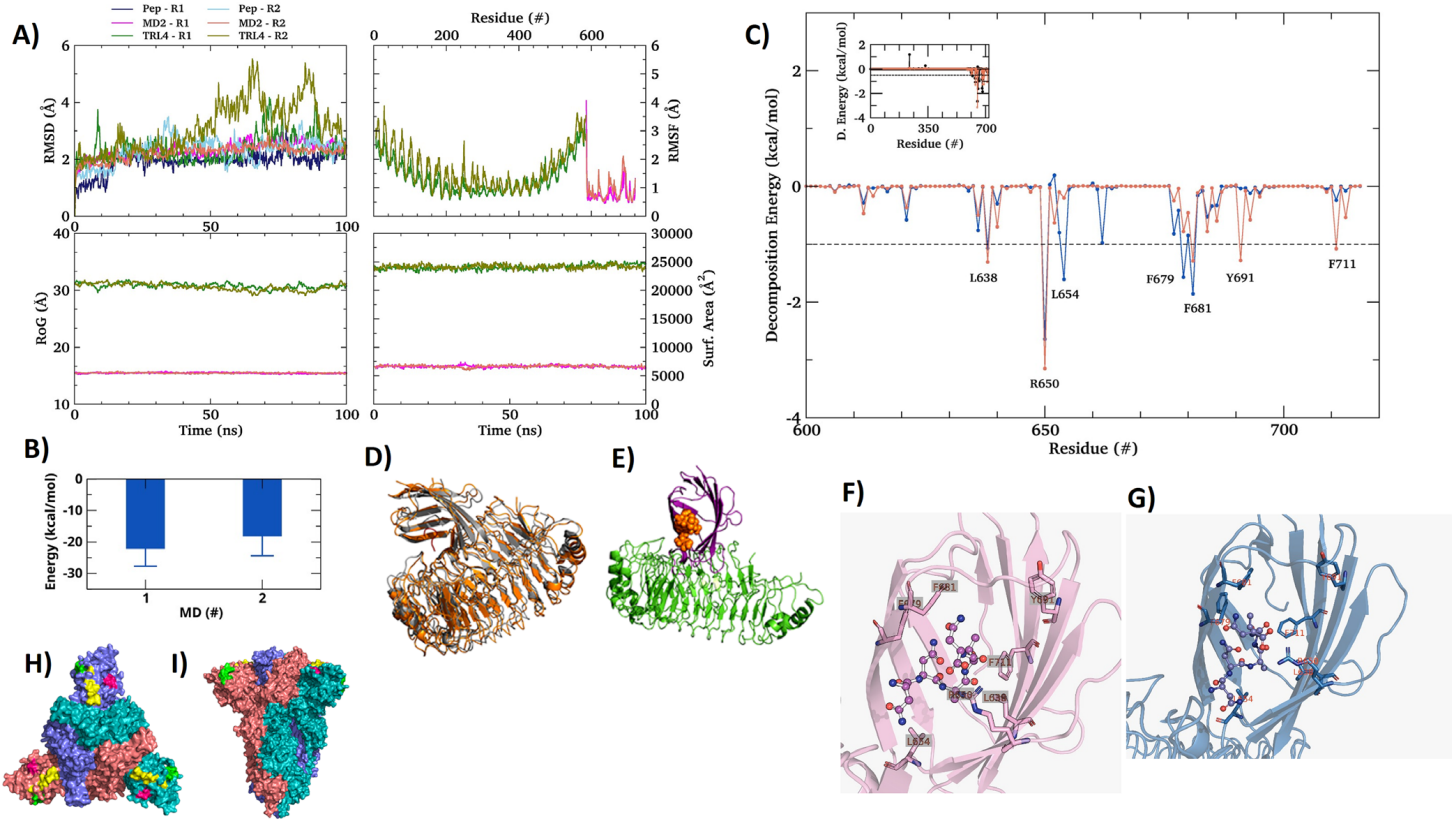
Molecular dynamics stability, binding energetics and structural features of the PSPD2003–TLR4 complex. (A) RMSD, RMSF, radius of gyration (RoG), and solvent‐accessible surface area (SASA) profiles from two independent MD replicates, indicating structural stability of TLR4 and expected flexibility of the peptide. (B) MM/GBSA binding free energy estimates for each replicate. (C) Per‐residue energy decomposition identifying key TLR4 residues contributing to peptide binding. (D, E) Superposition of MD trajectories with the initial structure, demonstrating consistent convergence of the peptide binding pose. (F, G) Close‐up views of the peptide‐binding site, highlighting the main stabilizing interactions. (H, I) Spatial distribution of selected SARS‐CoV‐2 spike protein‐derived peptides on the spike trimer.

The temporal stability of the PSPD2003–TLR4 complex was evaluated through extensive MD analyses using multiple structural metrics. RMSD and RMSF profiles (Figure 8A) demonstrated clear structural convergence of the TLR4 backbone, with RMSD values for the receptor (green and blue lines) remaining stable throughout the 100‐ns simulation. These results confirm that the tertiary structure of TLR4 was preserved and not disrupted by peptide binding. In contrast, PSPD2003 displayed the expected degree of flexibility for a short peptide, with RMSD values fluctuating between 4–5 Å after an initial ~20‐ns equilibration. These variations did not compromise complex stability.

RMSF analysis (Figure 8A, top right) further supported residue‐level stability, as most TLR4 residues exhibited low fluctuation amplitudes. The highest RMSF peaks corresponded to loop and turn regions (approximately residues 500–600), which are inherently dynamic and not directly involved in the binding interface. Additional global structural descriptors, including the radius of gyration (RoG) and solvent‐accessible surface area (SASA) (Figure 8A, bottom panels), showed consistent values across simulations, indicating the absence of large‐scale structural rearrangements or compaction/expansion events.

Binding affinity analyses provided strong support for a stable interaction. The calculated binding free energies (Figure 8B) were −22.1 ± 5.6 kcal/mol (MD1) and −18.1 ± 6.3 kcal/mol (MD2), values well within the range associated with high‐affinity molecular complexes. Per‐residue energy decomposition (Figure 8C) revealed the contribution of key TLR4 residues—L638, L654, R650, P681, Y691 and F711—which consistently exhibited favourable energetic contributions (*E* < 0). These residues cluster within the conserved 600–700 region of TLR4, confirming the specificity of the binding interface (Figure 8D–G).

Importantly, the functional relevance of this interaction was corroborated by in vivo assays, which validated that PSPD2003 exerts measurable biological effects consistent with TLR4 modulation.

Taken together, these high‐resolution structural and functional findings (Figure 8A–F) provide robust evidence that PSPD2003 acts as a stable, specific, and biologically active ligand of TLR4. The results support a plausible molecular mechanism through which SARS‐CoV‐2 Spike‐derived fragments may directly engage and activate innate immune signalling pathways, contributing not only to nociceptive processes but also to broader inflammatory disturbances associated with COVID‐19. Furthermore, the ability of PSPD2003 to modulate TLR4 activity positions this peptide as a promising molecular scaffold for the development of therapeutic antagonists aimed at mitigating TLR4‐mediated hyperactivation during SARS‐CoV‐2 infection.

### 3.8 Effect of PSPD2003 on Human Microglial Cells In Vitro

To further investigate the hypothesis that PSPD2003‐induced nociception may involve TLR4‐mediated microglial activation, we performed in vitro experiments using immortalized human microglial (C20) cells stimulated with PSPD2003, in the presence or absence of the TLR4 antagonist LPS‐RS.

**FIGURE 9:**
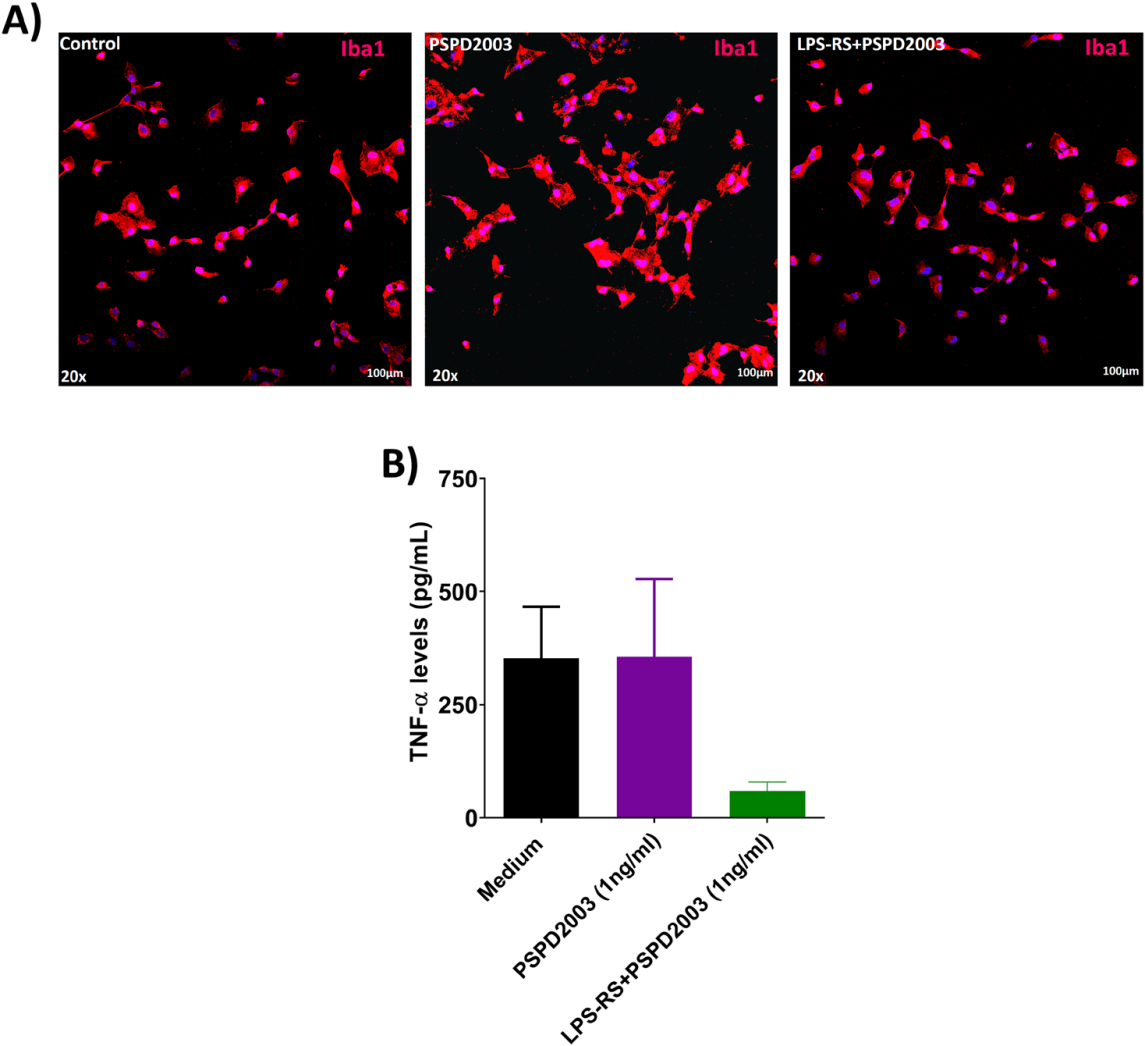
Involvement of TLR4 in PSPD2003‐induced microglial activation in vitro. (A) Representative confocal immunofluorescence images of human C20 microglial cells following 6 h of treatment with PSPD2003, culture medium alone (Control), or pretreatment with the TLR4 antagonist LPS‐RS for 2 h prior to PSPD2003 exposure. Cells were immunolabelled with an anti‐Iba1 antibody (red) to identify microglia and counterstained with DAPI (blue) to visualize cell nuclei. Representative images were selected from randomly acquired fields obtained from independent experiments. Images were acquired using a confocal fluorescence microscope at 20× magnification. Scale bar = 100 μm. (B) TNF‐α levels measured in the culture supernatant of human C20 microglial cells following 6 h of stimulation with PSPD2003, with or without pretreatment with LPS‐RS for 2 h. Data are presented as mean ± SD. Statistical analysis was performed using one‐way ANOVA followed by Bonferroni's multiple comparisons test.

Representative immunofluorescence images revealed that PSPD2003‐treated cells exhibited a hypertrophic morphology characterized by enlarged cell bodies, features commonly associated with microglial activation. In contrast, microglial cells from the control group, which received culture medium only, displayed a typical resting‐like morphology. Notably, pretreatment with LPS‐RS largely prevented the morphological changes induced by PSPD2003, preserving a phenotype similar to that observed in control cells (Figure 9A).

Analysis of TNF‐α levels in culture supernatants showed that PSPD2003 stimulation did not significantly increase cytokine production compared with the control group (Figure 9B). However, a trend toward reduced TNF‐α levels was observed in cells pretreated with LPS‐RS prior to PSPD2003 exposure. Although this reduction did not reach statistical significance, these findings, together with the morphological alterations observed by immunofluorescence, suggest that PSPD2003 may directly interact with microglial cells and promote cellular responses that are, at least in part, dependent on TLR4 signalling.

## 4 Discussion

The present study demonstrated that specific peptides derived from the SARS‐CoV‐2 Spike protein are capable of inducing nociceptive responses. In particular, we showed that nociception triggered by the synthetic peptide PSPD2003 involves the activation of spinal TLR4 and microglial cells. These findings provide new insight into how viral protein fragments may directly contribute to pain signalling pathways during SARS‐CoV‐2 infection.

Nociception has been consistently reported among individuals with COVID‐19. The prevalence of myalgia varies substantially across studies, ranging from 3.36% to more than 64% (Tsai et al. 2020), with a pooled estimated prevalence of approximately 19.3% among infected patients (Favas et al. 2020). Importantly, clinical evidence indicates that even non‐hospitalized patients may experience worsening of pre‐existing neuropathic symptoms for several weeks after infection, suggesting that pain exacerbation is not restricted to severe forms of COVID‐19 (Attal et al. 2021).

The emergence of SARS‐CoV‐2 mutations, particularly in the Spike protein, has been associated with changes in disease severity and symptom presentation, including pain. Because viral protein fragments may access peripheral and central nervous system compartments, structural changes in Spike‐derived peptides could alter receptor activation profiles, including TLR4 engagement, thereby modulating nociceptive signalling. In this context, our findings support the hypothesis that SARS‐CoV‐2 Spike‐derived peptides act as bioactive modulators of innate immune receptors, contributing to sensory disturbances reported during and after infection.

The SARS‐CoV‐2 S glycoprotein mediates viral entry into host cells and forms the characteristic crown‐like protrusions on the viral surface. During infection, the S protein interacts with host receptors, including TLR4, and undergoes proteolytic cleavage into the S1 and S2 subunits (Kirchdoerfer et al. 2016; Hoffmann, Kleine‐Weber, and Pöhlmann 2020; Shang et al. 2020; Conte 2021). These cleavage events, mediated by furin‐like or surface proteases, enable membrane fusion and viral internalization (Kirchdoerfer et al. 2016; Hoffmann, Kleine‐Weber, and Pöhlmann 2020; Shang et al. 2020).

Our findings demonstrated that the Spike‐derived peptides PSPD2001, PSPD2022 and PSPD2003 produced TLR4‐dependent nociceptive responses at the spinal level. TLR4 plays a critical role in spinal nociceptive processing and is implicated in multiple acute and chronic pain conditions (Lacagnina et al. 2018). Consistent with this role, PSPD2003 significantly increased spinal TLR4 mRNA expression, supporting direct receptor engagement.

In contrast, although PSPD2001 and PSPD2022 also induced TLR4‐dependent nociception, they did not alter receptor gene expression, suggesting indirect activation mechanisms or modulation of endogenous ligands converging on TLR4 signalling. These findings identify PSPD2003 as the most biologically active and direct TLR4 modulator among the peptides tested.

Previous studies have demonstrated that SARS‐CoV‐2 Spike‐derived regions can bind and modulate TLR4 signalling. Bhattacharya et al. (2020) identified four antigenic 9‐mer epitopes from the Spike protein that bind stably to the TLR4/MD‐2 complex. In silico studies also support direct binding of the full‐length Spike glycoprotein to TLR4 (Choudhury and Mukherjee 2020).

Importantly, although PSPD2003 demonstrated stable binding to TLR4 as an isolated peptide, this interaction may differ when the sequence is embedded within the intact Spike protein. Structural accessibility is influenced by conformational changes, domain organization, and proteolytic processing (Ke et al. 2020; Hoffmann, Kleine‐Weber, Schroeder, et al. 2020). Notably, the peptides investigated are derived from exposed regions of the S1 subunit, which may become accessible after proteolytic cleavage. In this context, Spike‐derived fragments could interact with TLR4 directly or through accessory molecules such as LBP, CD14 and MD‐2, contributing to innate immune activation (Bastos et al. 2023; Vabret et al. 2020).

These observations align with the present findings demonstrating that PSPD2003 is a stable and functionally active TLR4 ligand. Our data support the hypothesis that PSPD2003 directly engages TLR4 and may contribute to nociceptive manifestations associated with COVID‐19. Furthermore, its ability to modulate TLR4 signalling highlights its potential relevance for therapeutic strategies targeting infection‐associated hyperinflammation.

A complementary mechanism has also been described in which SARS‐CoV‐2 engagement with TLR4 promotes ACE2 upregulation, facilitating viral entry and amplifying inflammatory responses (Aboudounya and Heads 2021). Together, these findings strengthen the interpretation that spinal TLR4 contributes to the nociceptive responses induced by the evaluated peptides, particularly PSPD2003.

TLR4 signalling is mediated by Toll/Interleukin‐1 receptor (TIR) domains that recruit adaptor molecules such as MyD88 and TRIF. Activation of these pathways promotes transcriptional responses leading to the production of pro‐inflammatory cytokines involved in spinal nociceptive sensitization (Liu et al. 2022). Consistent with this mechanism, PSPD2003 significantly increased spinal TNF‐α and IL‐6 levels, both recognized mediators of neuroinflammatory responses.

Interestingly, PSPD2002 also increased spinal IL‐6 levels without affecting TNF‐α expression, whereas PSPD2001 did not significantly alter either cytokine. These findings further support the discussion above, suggesting that distinct Spike‐derived peptides may differentially engage inflammatory signalling pathways and activate partially divergent neuroimmune mechanisms.

Activation of glial cells through TLR4 represents a major mechanism driving the release of pro‐inflammatory cytokines involved in nociceptive sensitization (Acioglu et al. 2022). In agreement, pre‐treatment with the microglial inhibitor minocycline reversed PSPD2003‐induced nociception, supporting the involvement of microglial activation. Moreover, PSPD2003 markedly increased microglial reactivity in the SCDH of wild‐type and CX3CR1^GFP/+^ mice, whereas this effect was absent in TLR4^−/−^ mice, demonstrating TLR4‐dependent microglial activation. These in vivo findings were further supported by our in vitro experiments using immortalized human microglial cells. Exposure to PSPD2003 induced marked morphological changes characterized by a hypertrophic phenotype typically associated with microglial activation, whereas pretreatment with the TLR4 antagonist LPS‐RS largely prevented these alterations. Although PSPD2003 did not significantly increase TNF‐α release under the experimental conditions employed, the ability of TLR4 blockade to prevent the observed morphological changes provides additional evidence that PSPD2003 can directly engage microglial cells through a TLR4‐dependent mechanism.

In the present study, increased TMEM119 levels were observed following PSPD2003 administration, suggesting microglial involvement. However, the use of TMEM119 as a marker of microglial activation remains a matter of debate. Although TMEM119 is widely recognized as a microglia‐specific marker under physiological conditions (Bennett et al. 2016; Satoh et al. 2016), its regulation under inflammatory contexts is not uniform. Previous studies have demonstrated that TMEM119 expression can be differentially modulated depending on the nature of the stimulus, with reports of both upregulation and downregulation (Krasemann et al. 2017; Masuda et al. 2020). Notably, in models of systemic inflammation induced by LPS, TMEM119 expression has been shown to decrease (Zrzavy et al. 2017), indicating that its expression does not necessarily correlate directly with microglial activation status. Therefore, changes in TMEM119 levels should be interpreted with caution and ideally in combination with additional markers and functional approaches. Importantly, in the present study, the involvement of microglia is supported by complementary pharmacological and genetic evidence, rather than relying solely on TMEM119 expression.

Our observations align with previous reports showing that the SARS‐CoV‐2 Spike protein activates microglia through TLR4 signalling and promotes the production of pro‐inflammatory mediators (Frank et al. 2022; Jeong et al. 2022; Olajide et al. 2022; Samudyata et al. 2022). Together, these findings reinforce the notion that Spike‐derived fragments can engage innate immune pathways within the spinal cord and contribute to neuroinflammatory processes facilitating nociceptive transmission.

Microglial activation can occur through distinct receptor‐dependent signalling pathways, and different domains of the Spike protein appear to selectively engage these mechanisms. The recombinant full‐length S glycoprotein induces the secretion of inflammatory mediators, such as IL‐1 and CXCL8 through TLR4 signalling independently of ACE2, whereas the receptor‐binding domain selectively promotes IL‐18, TNF‐α and S100B release through ACE2‐dependent pathways (Tsilioni and Theoharides 2023). These findings demonstrate the domain‐specific functional organization of the Spike glycoprotein.

This mechanistic distinction may explain the lack of increased TLR4 mRNA expression observed after PSPD2001 and PSPD2002 administration. Although these peptides elicited TLR4‐dependent nociceptive responses, they may act through indirect or upstream modulators of TLR4 signalling, unlike PSPD2003, which appears to interact more directly with the receptor.

As previously reported, intracellular signalling cascades, such as p38 MAPK and NF‐κB, are key regulators of microglial activation and chronic pain states (Popiolek‐Barczyk and Mika 2016). In the present study, pharmacological inhibition of p38 MAPK and NF‐κB prevented PSPD2003‐induced nociception, indicating that activation of these pathways is required for its pronociceptive effects. Together, these findings support the involvement of a TLR4/microglia/p38–NF‐κB/cytokine signalling axis in PSPD2003‐induced nociception.

In contrast, pharmacological inhibition of p38 MAPK and NF‐κB did not alter PSPD2001‐ or PSPD2002‐induced nociception, suggesting that distinct signalling mechanisms may underlie the pronociceptive effects of these peptides. Previous evidence has implicated the canonical TIRAP/MyD88 signalling pathway in nociceptive processing and neuroinflammatory responses (Lacagnina et al. 2018; Dos Santos et al. 2020). Therefore, although the precise mechanisms triggered by PSPD2001 and PSPD2002 remain unclear, the involvement of alternative TLR4‐associated signalling cascades cannot be excluded.

This study has some limitations that should be considered when interpreting the findings. First, only male mice were included, which may limit the generalizability of the results, as accumulating evidence indicates that pain processing and neuroimmune signalling differ between sexes (Vacca et al. 2021; Barcelon et al. 2023; Ghazisaeidi et al. 2023). Male animals were selected to reduce biological variability and ensure experimental consistency; however, sex‐specific differences in PSPD2003‐induced nociceptive and neuroimmune responses cannot be excluded. Future studies including female animals with appropriate estrous cycle control will be important to determine whether the mechanisms identified here are sexually dimorphic.

Another limitation concerns the in vitro experiments. Although C20 human microglial cells provided complementary evidence supporting the involvement of TLR4 signalling in PSPD2003‐induced responses, an LPS‐RS‐alone control group was not included. This decision was based on the well‐established pharmacological profile of LPS‐RS as a competitive TLR4 antagonist that blocks receptor activation without triggering downstream inflammatory signalling in the absence of a pro‐inflammatory stimulus. Consistent with this mechanism, previous studies have demonstrated that LPS‐RS alone does not significantly alter the production of inflammatory mediators in microglial cultures, including TNF‐α, under basal conditions (Gaikwad and Agrawal‐Rajput 2015; Yang et al. 2018). Nevertheless, inclusion of this control group would have further strengthened the pharmacological characterization of TLR4 blockade in the present study.

An additional limitation is that PSPD2003 stimulation did not significantly increase TNF‐α levels in C20 microglial cells. Although this finding suggests that TNF‐α is unlikely to be a primary mediator of PSPD2003‐induced responses in isolated microglia, it does not exclude its contribution to the neuroinflammatory and nociceptive mechanisms observed in vivo. Neuroinflammation is orchestrated through dynamic interactions among microglia, astrocytes, oligodendrocytes and neurons, with neuroglial crosstalk amplifying and sustaining inflammatory signalling. As highlighted by Zong et al. (2026), glial cells form an interconnected and bidirectional network that actively regulates neuroinflammatory responses rather than functioning solely as passive support cells. Consequently, isolated C20 microglial cultures cannot fully recapitulate the complexity of the intact central nervous system, where TNF‐α production may result from coordinated signalling among multiple cell populations. Therefore, the absence of significant TNF‐α modulation in vitro should be interpreted with caution and should not be considered evidence against the involvement of TNF‐α or other glia‐dependent inflammatory mechanisms in PSPD2003‐induced nociception. Future studies using co‐culture systems, organotypic spinal cord preparations and broader profiling of inflammatory mediators will be important to better define the cellular and molecular pathways underlying PSPD2003‐induced neuroinflammation.

## 5 Conclusion

In conclusion, the present study is the first to demonstrate that the SARS‐CoV‐2 S protein can induce nociception through activation of spinal microglia and release of pro‐inflammatory cytokines via TLR4 signalling. These findings provide mechanistic insight into how viral protein fragments may directly engage innate immune pathways within the central nervous system and contribute to pain manifestations associated with COVID‐19. Moreover, our results identify TLR4 as a promising therapeutic target and highlight the potential of TLR4‐modulating strategies for managing infection‐associated pain and hyperinflammatory responses triggered by SARS‐CoV‐2.

## Author Contributions

B.E.S., R.S.S. and G.G. conceived the study. F.P.V., R.R.C., L.M.R.R., E.M.C., D.O. and M.A.A.B. were involved with data acquisition. G.G., R.S.S., B.E.S. and D.O. performed data analysis, image analysis and image quantification. B.E.S., D.O., G.G. and R.S.S. created the final figures. B.E.S., E.S.C., A.R.B., A.N.‐S., T.R.L.R., M.S., G.G. and R.S.S. were involved with behaviour and biomolecular data analysis. E.M.C., D.O., M.A.A.B. and I.C.‐S. are involved with molecular dynamics analysis. G.G. and R.R.S. wrote the initial article. All authors reviewed, edited and contributed significantly to the article. All authors approved the final version of the article.

## Funding

The study was supported by the Coordination for the Improvement of Higher Education Personnel (CAPES) [grant no. 001], the National Council for Scientific and Technological Development (CNPq) [grant no. 310467/2023‐3] and the Minas Gerais Research Foundation (FAPEMIG) [grant no. BPD‐00749‐22].

## Disclosure

Use of Artificial Intelligence: AI was not used in this manuscript.

## Conflicts of Interest

The authors declare no conflicts of interest.

## Supporting information


**Appendix S1:** ARRIVE guidelines: A checklist of 10 essential checklist items for animal studies.

**Appendix S2:** Primary sequence of the SARS‐CoV‐2 Spike glycoprotein and localization of the selected peptides.

**Appendix S3:** Schematic representation of the experimental protocol used to evaluate the spinal effects of SARS‐CoV‐2 S‐derived peptides on mechanical nociceptive thresholds. Baseline latency (BL) of nociceptive thresholds were first determined for each animal, followed by intrathecal administration of the peptides (PSPD2001, PSPD2002 or PSPD2003). Mechanical thresholds were subsequently reassessed at 1, 3, 5, 7 and 24 h post‐injection. To investigate the involvement of microglia, TLR4, p38 MAPK and NF‐κB signalling, selective antagonists or inhibitors (minocycline, LPS‐RS, SML0543 or PDTC) or their respective vehicles (0.9% sterile saline or 2% DMSO) were administered intrathecally 10 min prior to peptide injection.

**Appendix S4:** Images of the spinal cord sections from the respective experimental groups shown in Figure 7 that were used for the quantitative analysis of the number of TMEM119‐positive or CX3CR1‐positive microglia.
