# Supplementary material for: SARS‐CoV‐2 Spike Peptides Trigger Nociceptive Responses Through Spinal TLR4 Pathways
Source: Eur J Pain. 2026 Aug 4;30(7):e70343. doi: 10.1002/ejp.70343 (PMC13435826; doi:10.1002/ejp.70343)
Supplement: Supplementary file 1 [file EJP-30-0-s002.pdf]

Supplementary material 4

Images of microglial assessment using the TMEM119 marker in wild-type mice following PSPD2003 or saline administration.

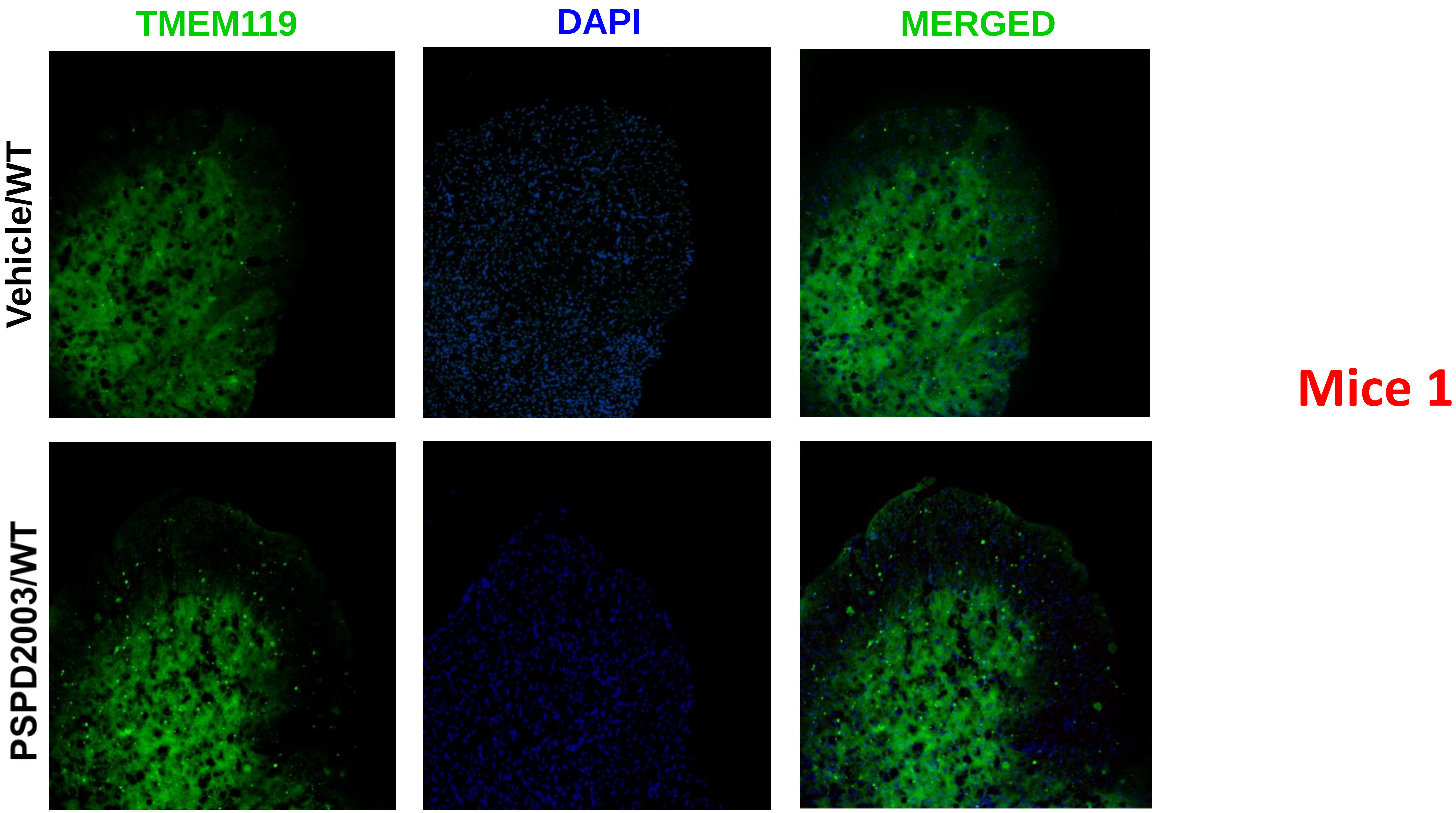

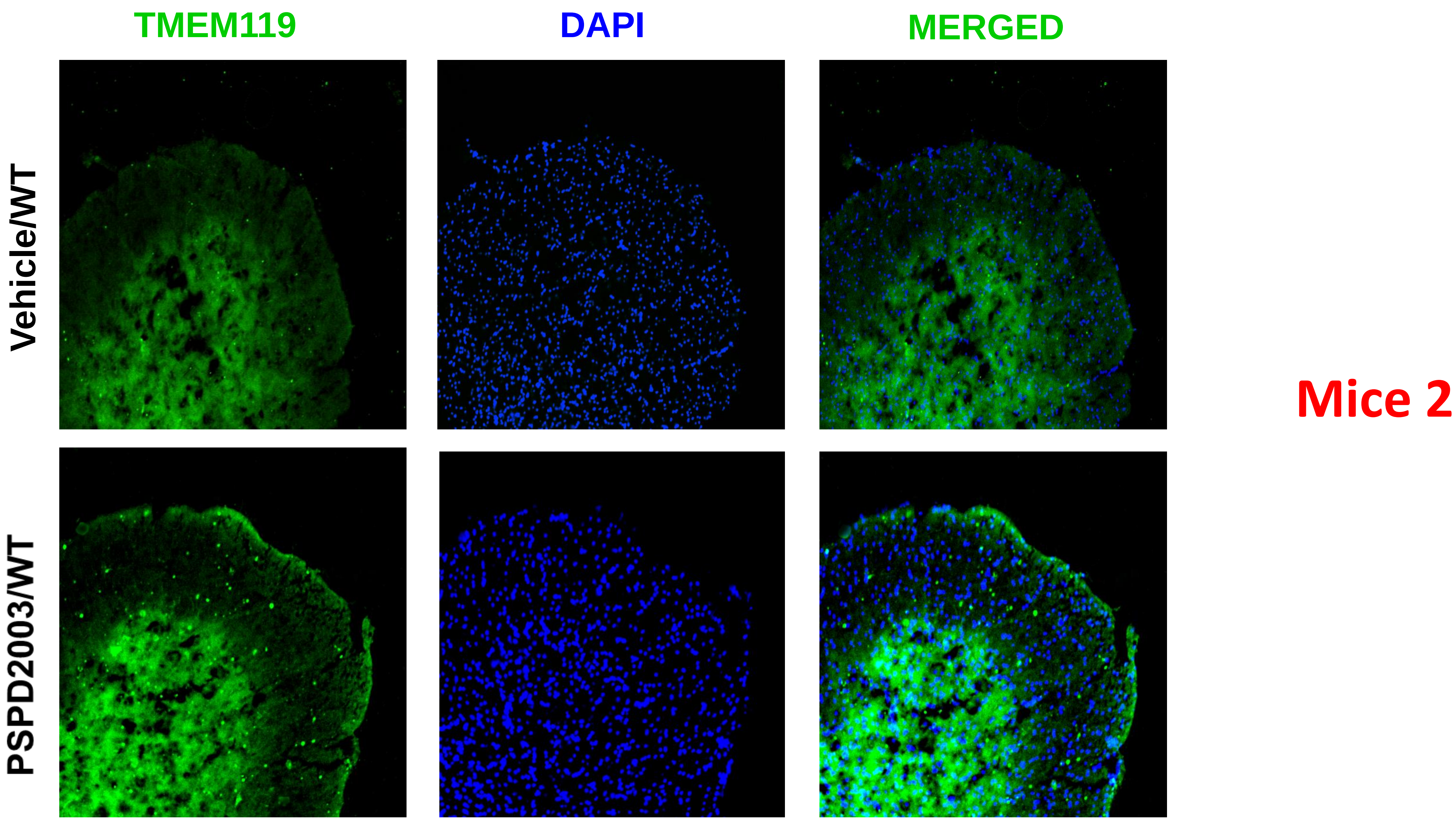
TMEM119
DAPI
MERGED
Vehicle/WT
PSPD2003/WT
Mice 2

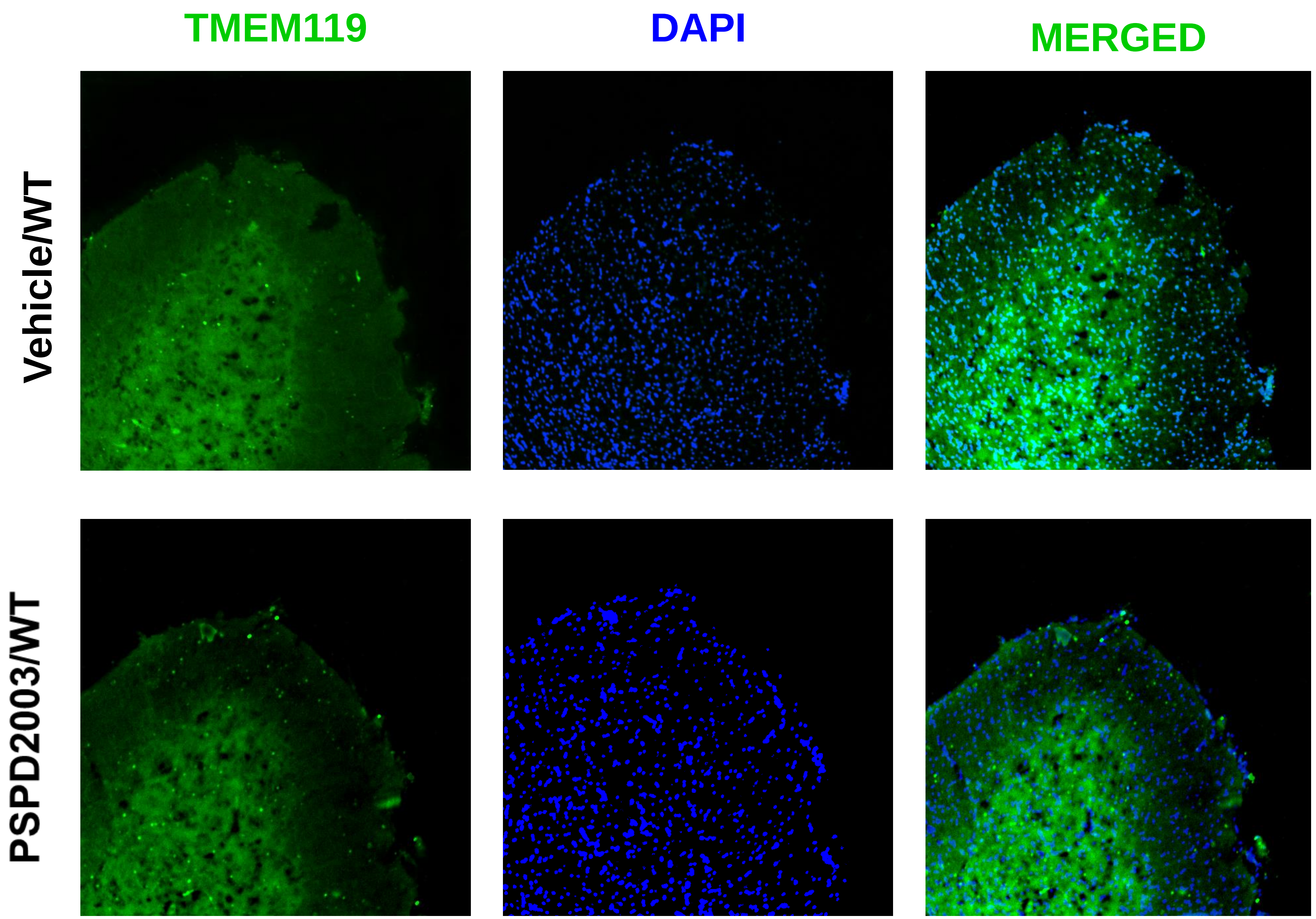

Mice 3

Images of microglial assessment in CX3CR1$^{GFP/+}$ mice following PSPD2003 or saline administration.

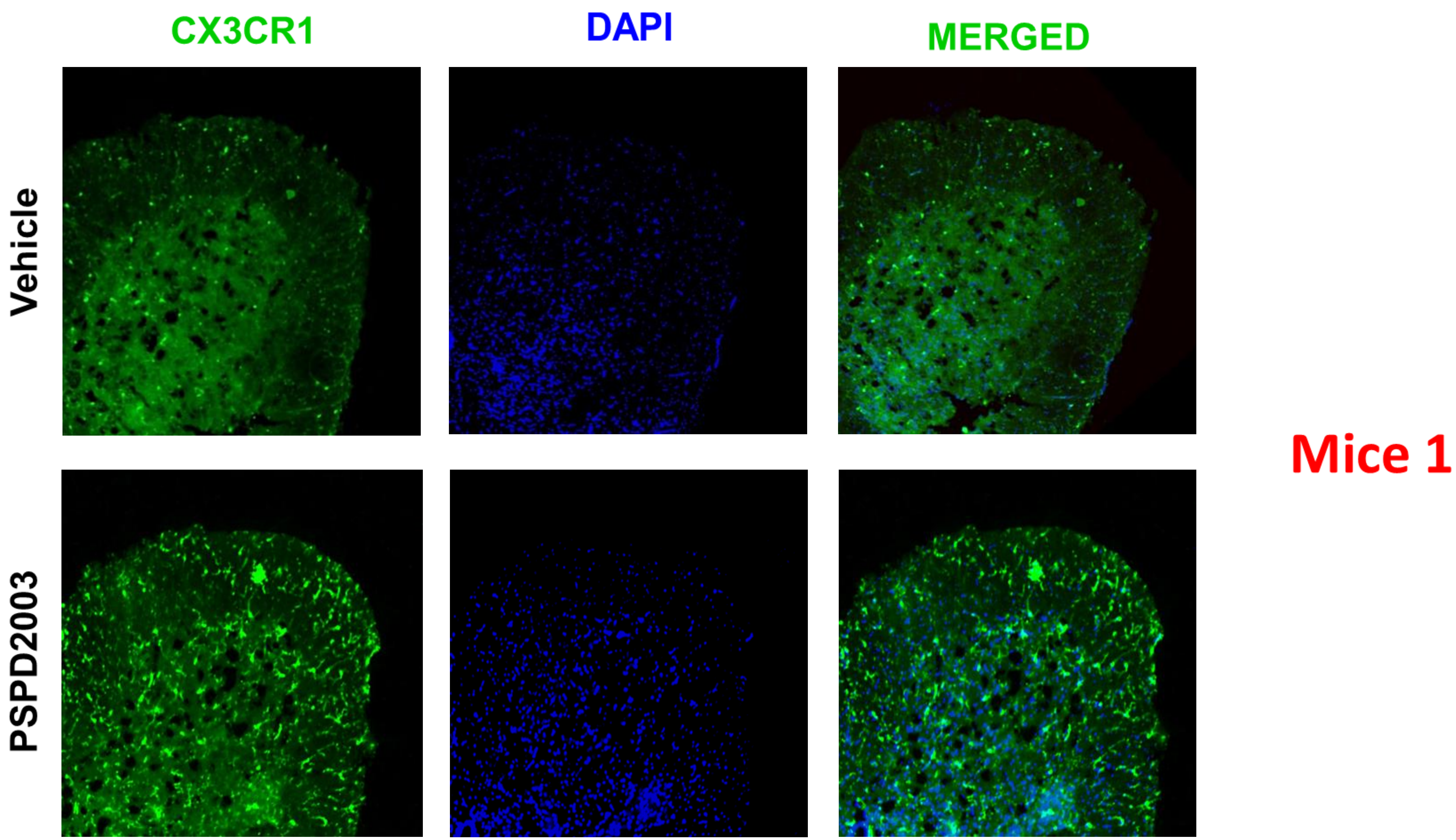

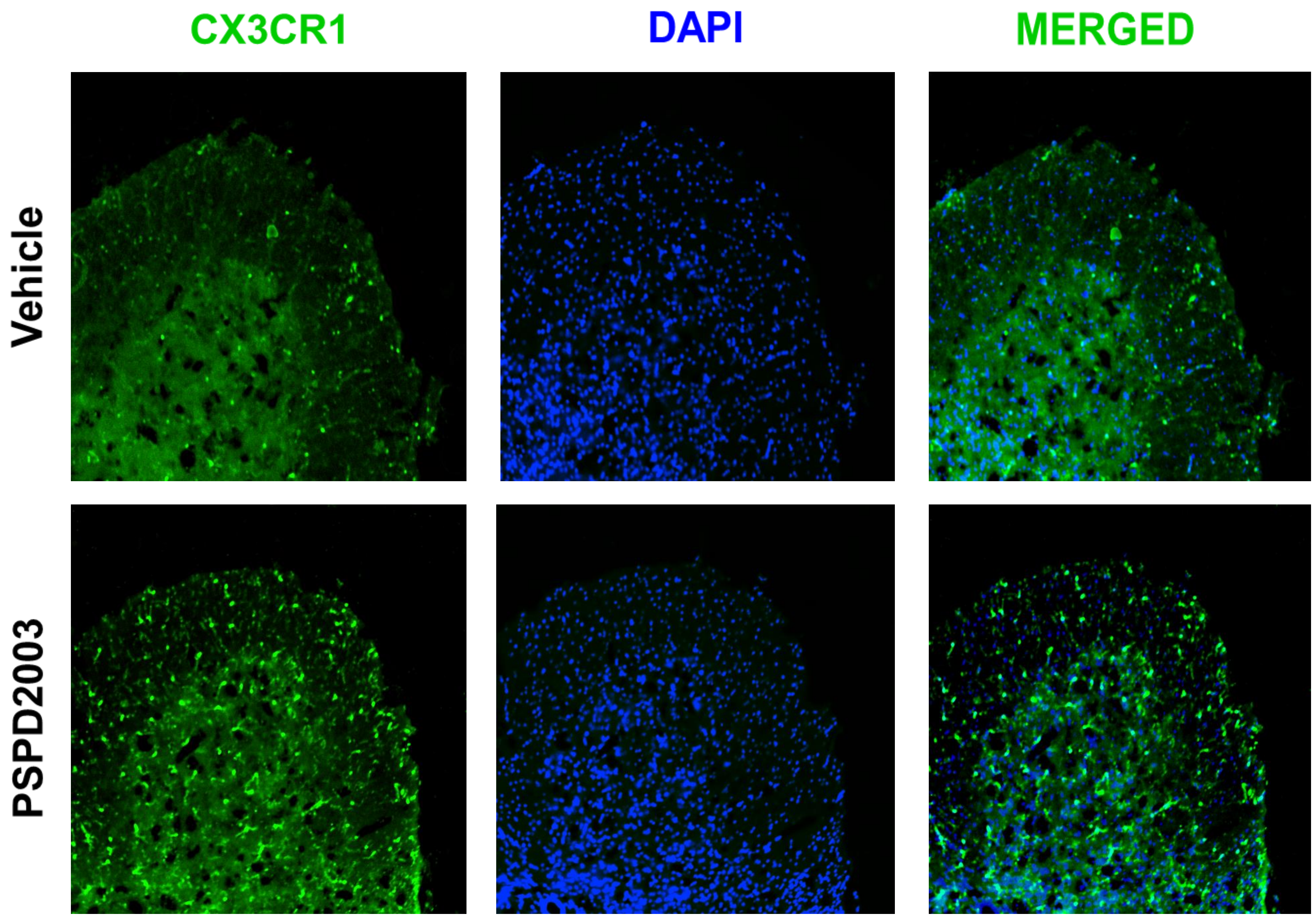

Mice 2

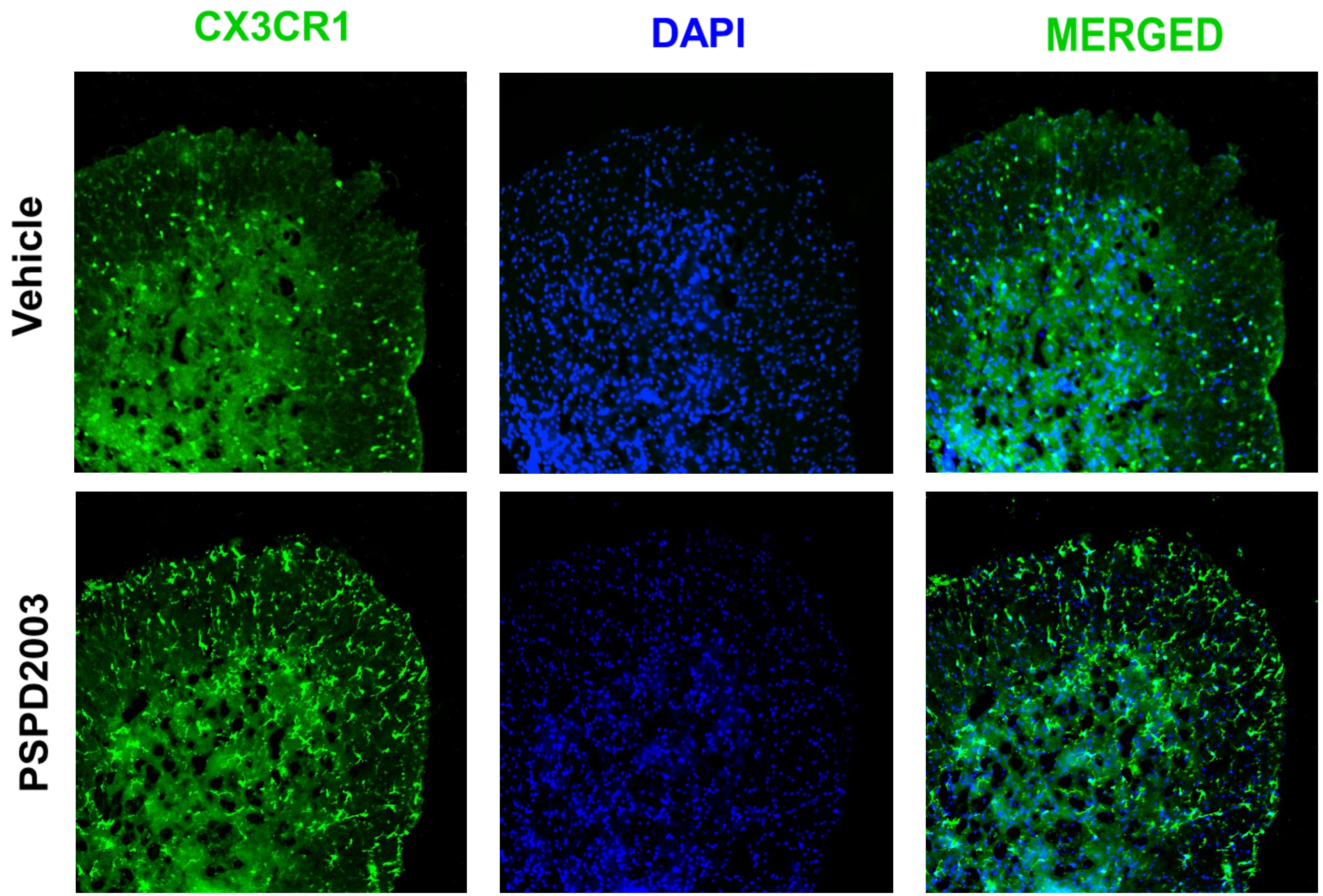

Mice 3

Images of microglial assessment using the TMEM119 marker in TLR4$^{-/-}$ mice following PSPD2003 or saline administration.

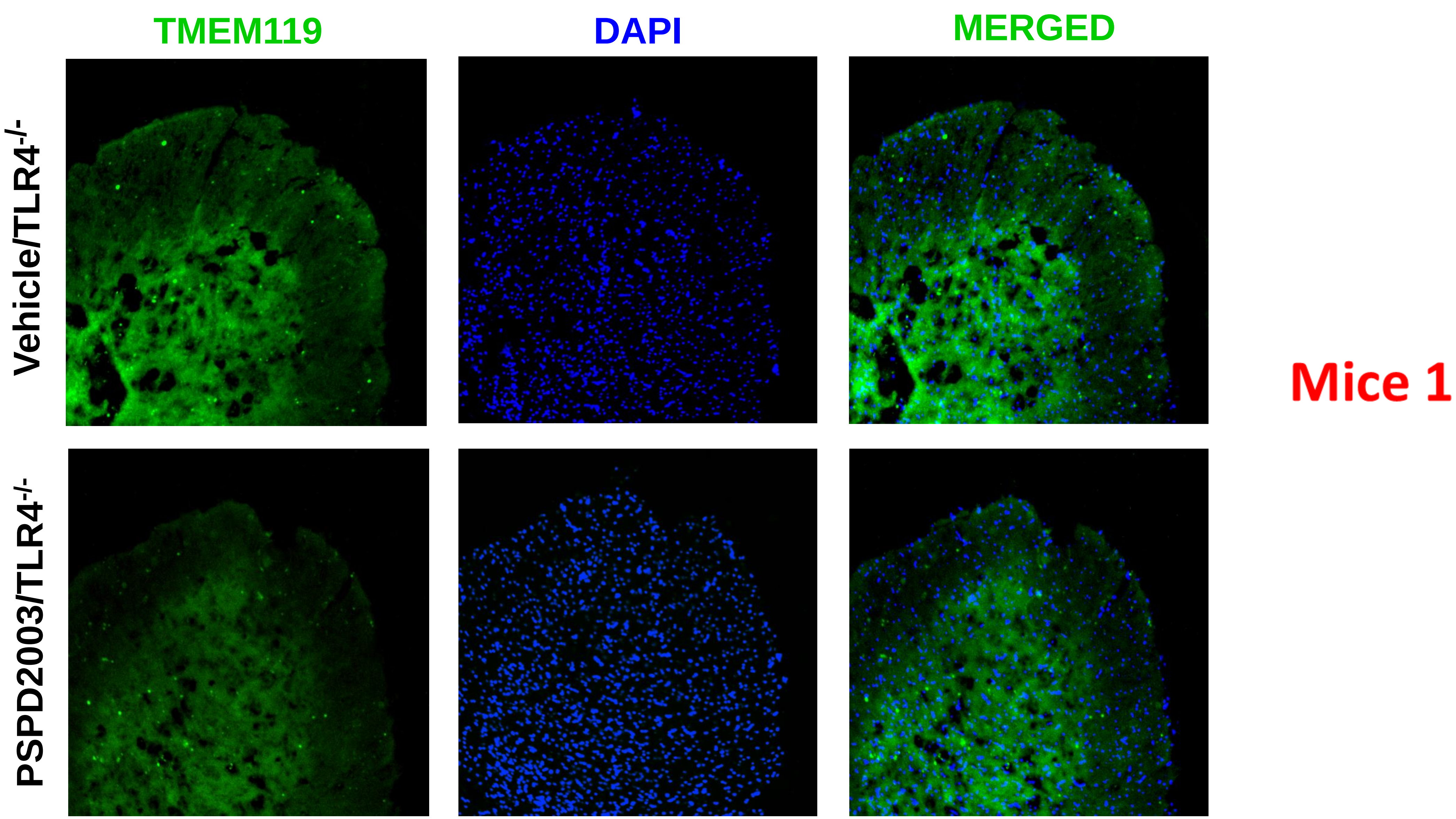

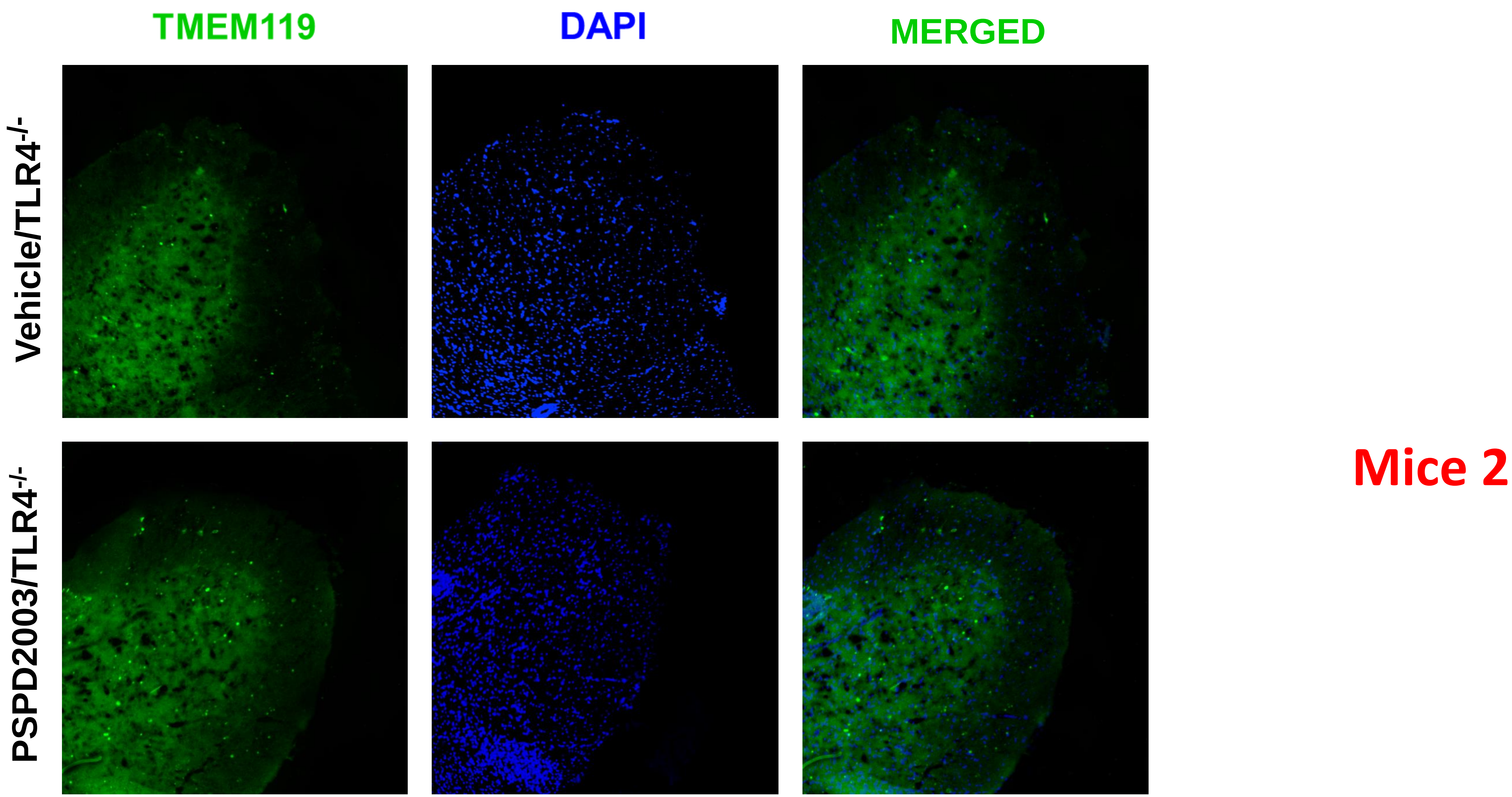
TMEM119
DAPI
MERGED
Vehicle/TLR4-/-
PSPD2003/TLR4-/-
Mice 2

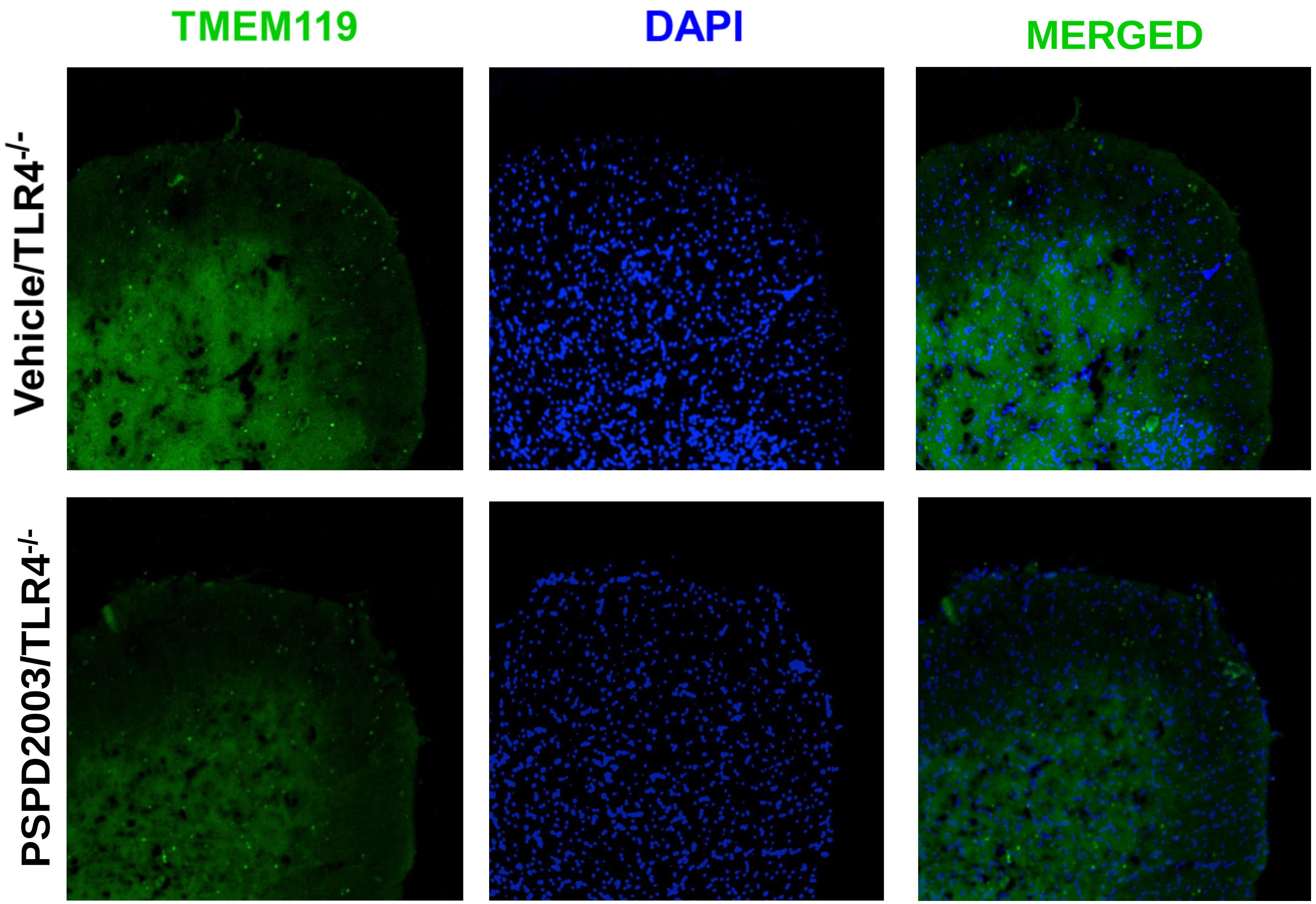

Mice 3
